# Antioxidant and Antidiabetic Activity of Algae

**DOI:** 10.3390/life13020460

**Published:** 2023-02-07

**Authors:** Atef Mohamed Abo-Shady, Saly Farouk Gheda, Gehan Ahmed Ismail, João Cotas, Leonel Pereira, Omnia Hamdy Abdel-Karim

**Affiliations:** 1 Botany Department, Faculty of Science, Tanta University, Tanta 31527, Egypt; 2 MARE—Marine and Environmental Sciences Centre/ARNET—Aquatic Research Network, Department of Life Sciences, University of Coimbra, Calçada Martim de Freitas, 3000-456 Coimbra, Portugal; 3 Bioprocess Engineering & AlgaePARC, Wageningen University and Research, 6700 AA Wageningen, The Netherlands

**Keywords:** diabetes, antioxidant, antihyperglycemic, lipid profile, body weight, algal treatments

## Abstract

Currently, algae arouse a growing interest in the pharmaceutical and cosmetic area due to the fact that they have a great diversity of bioactive compounds with the potential for pharmacological and nutraceutical applications. Due to lifestyle modifications brought on by rapid urbanization, diabetes mellitus, a metabolic illness, is the third largest cause of death globally. The hunt for an efficient natural-based antidiabetic therapy is crucial to battling diabetes and the associated consequences due to the unfavorable side effects of currently available antidiabetic medications. Finding the possible advantages of algae for the control of diabetes is crucial for the creation of natural drugs. Many of algae’s metabolic processes produce bioactive secondary metabolites, which give algae their diverse chemical and biological features. Numerous studies have demonstrated the antioxidant and antidiabetic benefits of algae, mostly by blocking carbohydrate hydrolyzing enzyme activity, such as α-amylase and α-glucosidase. Additionally, bioactive components from algae can lessen diabetic symptoms in vivo. Therefore, the current review concentrates on the role of various secondary bioactive substances found naturally in algae and their potential as antioxidants and antidiabetic materials, as well as the urgent need to apply these substances in the pharmaceutical industry.

## 1. Introduction

The prevalence of diabetes has increased rapidly over the past few years, mainly in low- to middle-income countries, and has become one of the major causes of premature death worldwide. According to International Diabetes Federation (IDF) [1], there are about 537 million individuals living with diabetes, and about 316 million persons suffer from weakened glucose tolerance and augmented risk of diabetes. These statistics are predicted to increase to 643 million by 2030, and in less than a quarter of a century, it is predicted that there will be 783 million people suffering from this disease in the absence of rapid and accurate prevention procedures [1].

Diabetes mellitus is an extended metabolic disorder of several etiologies, characterized by chronic hyperglycemia with the ailment of carbohydrate, fat, and also protein metabolism, which typically results from an absolute or relative lack of insulin, impaired effectiveness of insulin action, or tissue insensitivity to insulin [2].

Insulin is a hormone produced by the pancreatic β-cells that functions to maintain the strict control of blood glucose. This hormone enables the tissues and cells of the body to utilize glucose for energy. If insulin is absent or its action is impaired due to tissue insensitivity, cells, and tissues are unable to uptake glucose, which results in its accumulation in the blood, and consequently, diabetes symptoms occur [3].

Symptoms of diabetes often go unobserved because they can be attributed to many other causes, and some patients fail to notice cautioning signs or practice definite indicative symptoms [4]. However, possible symptoms of diabetes may include the following: unexplained weight loss, excessive thirst (polydipsia), excessive urination (polyuria) and dehydration, excessive hunger, general fatigue, blurred vision, nearsightedness or other vision problem, and vaginal infection. The control of hyperglycemia (abnormally high glucose) is very important in the treatment of all forms of diabetes because, in the long term, acute and chronic complications can happen when the blood glucose concentration is not normalized [2].

**Figure 1:**
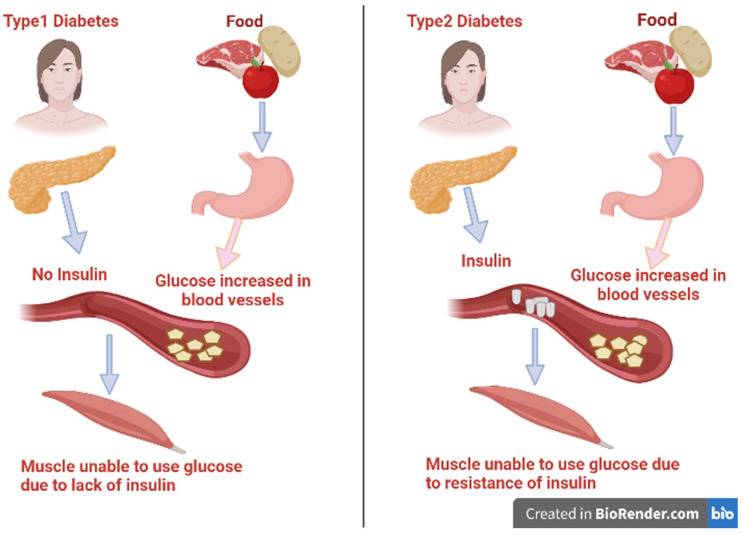
Types of diabetes mellitus and their syndrome.

There are various categories of diabetes: type 1 diabetes, type 2 diabetes, gestational diabetes mellitus*,* and other specific categories of diabetes that have several causes, such as pancreatic or drug-related diseases, monogenic diabetic syndrome, and chemical inducers [5]. The two main types of diabetes mellitus are illustrated in Figure 1. Type 1 diabetes is also identified as insulin-dependent diabetes mellitus (IDDM). It represents about 10% of all cases of diabetes and was previously known as juvenile-onset diabetes as it usually occurs in persons under 40 years of age [6,7]. In this type, there is usually a lack of the secretion of insulin as a result of disorders affecting and deteriorating the pancreatic β-cells. It is conveyed to have a genetic inheritance tendency and an autoimmune basis that lead to β-cell destruction due to the presence of anti-insulin antibodies [5]. As a result of insulin insufficiency, the body will be forced to burn fats for energy instead of glucose, resulting in a toxic byproduct called ketones under severe hyperglycemia [8]. Patients of IDDM need a daily dosage of insulin to live and avoid the progress of ketoacidosis.

Alternatively, type 2 affects about 95% of people diagnosed with diabetes mellitus and are 40 years and above in age [9]. It happens as a result of the progressive loss of β-cells secreting insulin or tissue insensitivity to absorb insulin that impairs insulin action [10]. Fast food globalization, unhealthy eating patterns, and inactivity could lead to an increase in body mass, and these may be the main causes of diabetes. Therefore, this review’s goal is to provide a thorough investigation of the bioactive extracts derived from micro- and macroalgal species and how they can aid in the treatment and/or prevention of complications associated with diabetes mellitus.

## 2. Factors That Contribute to Diabetes and Its Complications

**Figure 2:**
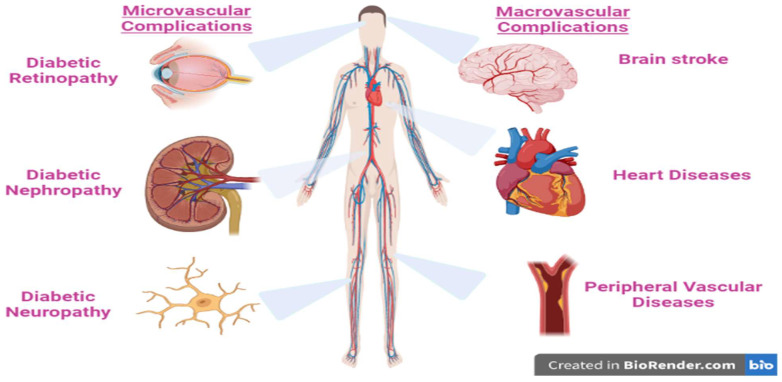
The major complication of type 2 diabetes mellitus.

The majority of diabetes types across the world may be correlated to modern diets, sedentary lifestyles, and obesity. The mortality associated with diabetes is mainly a result of the augmented danger of several complications of this disease. Even though diabetes is primarily defined by chronic hyperglycemia, many diabetic patients, particularly those with type 2, have elevated blood pressure (hypertension), chronic high levels of insulin (hyperinsulinemia), and abnormal levels of cholesterol, triglycerides, and/or other blood lipids (hyperlipidemia). In addition, lipoprotein abnormalities are some of the most prevalent problems associated with type 2 diabetes [11]. These complications are strictly related to the disease disorders, as well as to the procedures used to diagnose and treat them (Figure 2).

### Oxidative Stress Linked to Diabetes

**Figure 3:**
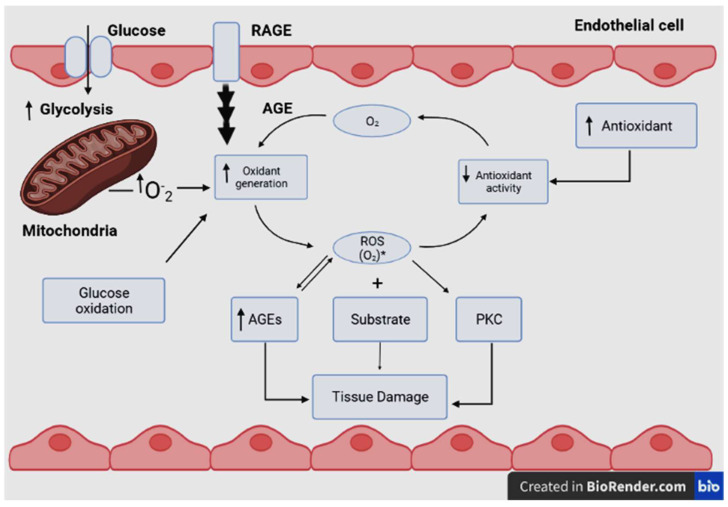
The relationship between rates of oxidant generation, antioxidant activity, oxidative stress, and oxidative damage in diabetes. O_2_^•−^ represents various forms of ROS. The overall rate of formation of oxidative products, which lead to oxidative tissue damage, is dependent on ambient levels of both O_2_^•−^ and substrate. Increased generation of O_2_^•−^ depends on several sources, including glucose autoxidation, increased mitochondrial superoxide production, and increased endoplasmic reticulum superoxide production, as well as the result of the receptor for advanced glycosylation end product activation. O_2_^•−^ deactivation is reduced because antioxidant defenses are compromised in diabetes. Note that oxidative stress also promotes other hyperglycemia-induced mechanisms of tissue damage. Additionally, oxidative stress activates protein kinase C (PKC) and accelerates the formation of advanced glycosylation end products (AGEs), RAGE: Receptor for AGEs.

Biochemical processes in the body may produce intermediate products called reactive oxygen species (ROS). Among ROS, harmful free radicals have one or more unpaired electrons that make them very reactive with other molecules. Excess ROS may lead to an imbalance in its metabolism and in the body’s ability to detoxify or counteract the harmful oxidant effects of the free radicals [12,13]. This condition is known as oxidative stress, which depends on the balance between ROS production and antioxidant defenses [14]. Oxidative stress is thus the result of the imbalance between the formation and neutralization of reactive oxygen and nitrogen species [15,16]. Electron transfers to O_2_ are catalyzed by oxidase enzymes to produce chemical energy or oxidation of substrates. These enzymes are potential sources of reduced Cu_2_^+^ derivatives in biological settings; they also produce O_2_^•−^ during catalysis [17,18,19]. The mitochondrial electron transport chain reduces O_2_ to O_2_^•−^ [20,21]. Dismutase enzymes reduce O_2_^•−^ radicals to form hydrogen peroxide (H_2_O_2_) and/or further react to form the hydroxyl secondary radical (^•−^OH) as another type of the ROS [17,19]. Although the cause-effect relationship remains unsure, there appears to be a strong correlation between mitochondrial dysfunction and chronic metabolic diseases, such as obesity [17] and diabetes mellitus (Figure 3) [22]. This relationship results in oxidative damage to cellular components in the form of lipid peroxidation, protein denaturation or DNA conjugation, and finally, cell death [23]. As a result of the aforementioned reasons, oxidative stress has been related to many diseases, such as cancer, post-ischemic and neural degradation, Parkinson’s and Alzheimer’s diseases, acquired immune deficiency syndrome (AIDS), aging, and cardiovascular diseases [24].

## 3. Treatment of Diabetes

The treatment of diabetes requires changes in lifestyle patterns and necessitates lifelong adaptations. In order to prevent, delay, or stop the microvascular and macrovascular effects of diabetes, the diabetic patient should be provided with the necessary tools to achieve the best management of glycemia, blood pressure, and lipidemia [25]. Currently, the available medicine for treating type 2 diabetes works by stimulating and augmenting endogenous insulin production at target tissues, as well as blocking the key enzymes that interfere with type 2 diabetes mellitus, such as α-amylase and α-glucosidase enzymes [26]. In the human body, these carbohydrate-mediated enzymes work synergistically to breakdown starch using pancreatic α-amylase and absorb glucose via intestinal α-glucosidase [27,28]. α-amylase is the main enzyme that regulates the digestion rate of starch through the hydrolysis of inner α-1,4-glucosidic linkages and forms linear or branched malt-oligosaccharides. α-glycosidase performs a role in the conversion of oligosaccharides into glucose, and this may lead to postprandial hyperglycemia (after-meal). Reducing postprandial hyperglycemia levels is the furthest functional therapeutic process for the inhibition of pancreatic α-amylase and α-glucosidase, which significantly delays carbohydrate digestion and glucose absorption with smaller consequences than the former diabetic treatments [7,29,30].

Some synthetic inhibitors of these enzymes are used clinically to manage or remedy diabetes, such as sulfonylureas, biguanide, glycosidase inhibitors, insulin, aldose reductase inhibitor, thiazolidinediones, carbamoyl methyl benzoic acid gliclazide, metformin, acarbose, and voglibose [31]. Continuous use of these synthetic agents should be limited because they may cause flatulence, abdominal cramps, vomiting, diarrhea, weight gain, nausea, upset stomach, and liver function disorders [32,33]. Increased efforts are being made to find and investigate potential inhibitors of α-glucosidase and α-amylase from natural sources to develop compounds for in vitro and in vivo usage as antidiabetic medications that show no side effects [34].

### 3.1. Antioxidants

Antioxidants are constituents that delay or avoid the oxidation process by neutralizing the free radicals scavenging in body cells [35]. The main source of antioxidants in the human body is vegetables and fruits in our diet [36,37]. There are countless commercial synthetic antioxidants, for example, butylated hydroxyl toluene (BHT), propyl gallate (PG), butylated hydroxy anisole (BHA), and tert-butylhydroquinone (TBHQ), which are used to reduce the harmful effects of free radicals. But these synthetic drugs may cause other harmful side effects [38], as their use in food products has been failing due to their instability or their suspected action as promoters of carcinogenesis [39]. Therefore, the search to replace these artificial antioxidants with novel resources of natural antioxidants has become a critical exploit in immunopharmacological research [39].

Natural antioxidants derived from natural sources can react rapidly with free radicals and impede or alleviate the extent of oxidative deterioration [40]. Furthermore, natural antioxidants can help inincreasing the food’s shelf life. Therefore, the consumption of natural antioxidant materials could protect the body and foods against these actions [41]. Recent studies suggest a contrary relationship between the dietary intake of antioxidant-rich foods and the incidence of human disease. atural antioxidant materials are presumed to have several positive health effects, including the prevention of cardiovascular disorders and certain cancers and possibly decreased mutation potentialities [42]. Moreover, they may be a dynamic, safe, and economical substitutional remedy for diabetes management and organ protection [43]. Natural antioxidants, such as tocopherols, ascorbic acid, carotenoids, flavonoids and related polyphenols, α-lipoic acid, glutathione, phlorotannins, and alkaloids are commonly known [44]. Fortunately, the majority of these compounds are natural components produced by plants and different algal species as secondary metabolites.

### 3.2. Algal Extracts and Their Bioactive Components Linked to Diabetes Treatment

Algae represent a great group of diverse organisms from dissimilar phylogenetic groups and also several taxonomical divisions. Generally, algae may be identified as plant-like organisms that are regularly photosynthetic and aquatic but without real roots, stems, leaves, and vascular tissues, besides having elementary reproductive structures. They are distributed worldwide in seawater, freshwater, soil, and wastewater [45]. According to their forms and sizes, algae have two major categories: single microscopic cells or multicellular organisms that live in colonies (microalgae), and macroscopic multicellular organisms (macroalgae). Cyanobacteria, previously known as blue-green algae, are morphologically various divisions of prokaryotic and photosynthetic organisms that flourish in varied types of habitats. Most species of cyanobacteria are free-living in freshwater, marine, or terrestrial habitats and are symbioses with other plants and lichens [46]. Algal organisms exhibited a wide diverse of cell sizes. Picoplanktonic algae are between 0.2 to 2 µm in diameter, while the fronds of giant kelps are as large as 60 m in length, with a leafy appearance [47].

The term seaweed refers to the large visible macroalgae growing and attaching to rocks along the seashore. Seaweeds have been used widely for multiple applications, such as human food, animal feeds, and fertilizers [48]. They can grow extensively in shallow marine water and estuaries. Seaweeds are scientifically classified as Rhodophyta (red algae), Phaeophyceae (brown algae), and Chlorophyta (green algae). This classification is based on pigment content, nutrients, chemical composition, morphological characteristics, and anatomical features [49].

Natural products derived from different algal species, such as alkaloids, flavonoids, terpenoids, steroids, and phenols, have received considerable attention over the years due to their diverse pharmacological properties, including their antioxidant and antidiabetic functions [50]. Among these compounds, alkaloids have cytotoxic activity by inhibiting the formation of the mitotic spindle fibers required for cell division [51]. Terpenoids display a wide spectrum of antitumor activities [52]. Steroids are recognized for having antimicrobial and cardiotonic properties, which play a vital role in nutrition, herbal medicine, and cosmetics [53]. Tannins are used therapeutically as antiviral, antibacterial, antiulcer, and antioxidant agents, as well as for inflammation as cytotoxic agents [54].

Likewise, flavonoids have antimicrobial, antioxidant, or free radical scavengers and spasmolytic activity [55]. Other biological compounds, such as saponins, are used in hypercholesterolemia and hyperglycemia as antioxidants and as anticancer, anti-inflammatory, and as weight-loss drugs [56]. Phenolic compounds are major bioactive components that were determined in the extracts of different algae [57,58] and proved to exert significant antioxidant activities. These are a large chemical group that associated with the defense mechanisms against endogenous and exogenous environmental effects, such as oxidative processes, light, temperature, and pathogen invasion [59].

### 3.3. Antioxidant and Antihyperglycemic Activity of Different Algal Extracts

**Figure 4:**
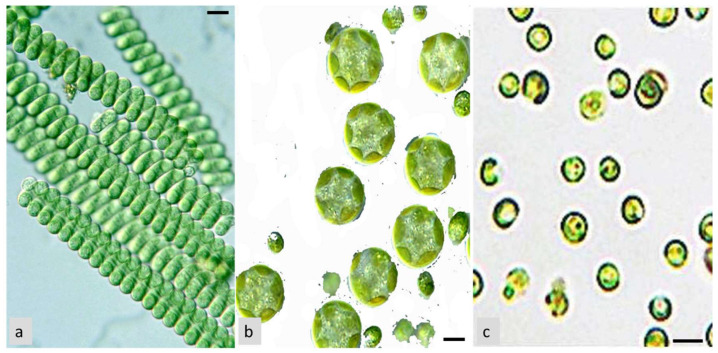
Microalgae species images: (**a**)—*Arthrospira platensis* (Cya); (**b**)—*Chlorella vulgaris* (Chl); (**c**) *Nannochloropsis* (Eus); (Cya)—Cyanobacteria; (Chl)—Chlorophyta; (Eus)—Eustigmatophyceae. Scale = 10 μm.

The therapeutic potential of microalgae was documented by many studies to be associated with their biological compounds, such as alkaloids, carotenoids, terpenoids, steroids, phlorotannins, phenolic compounds, halogenated ketones, alkanes, cyclic polysulfides, in addition to being rich in proteins, vitamins, pigments, lipids, and essential minerals [60]. Butanol extract from *Arthrospira platensis* cyanobacterium (formerly *Spirulina platensis*) (Figure 4a) showed the maximum antioxidant activities with DPPH (2,2-diphenyl-1-picrylhydrazyl), reducing power, hydroxyl radical, and nitric oxide scavenging activity assays [61]. In the same manner, Jayshree et al. [62] reported that *Chlorella vulgaris* (Chlorophyta) (Figure 4b) methanolic extract showed antioxidant activity in terms of DPPH radicals scavenging, phosphomolybdate and reducing power assays. Also, the pronounced DPPH scavenging property of the aqueous extracts of *Arthrospira* sp. and *Nannochloropsis* sp. (Ochrophyta, Eustigmatophyceae) (Figure 4c) was recorded by Scaglioni et al. [63]. In addition, the acetone extract of *C. vulgaris* showed antioxidant activity using different standard assays due to the presence of active secondary metabolites [64]. Recent research found that the microalgae *Nannochloropsis* sp. had a higher concentration of essential fatty acids and demonstrated potential antioxidant activities for the methanolic extract, demonstrating that it could be used as a natural antioxidant and help prevent oxidative stress [65]. Additionally, Gheda et al. [57] informed that the methanol extract of *A. platensis* exhibited the highest antioxidant activity for all tested assays (DPPH, reducing power, and total antioxidant capacity assays). Moreover, the phytochemical components in the different solvents of *Hydrodictyon reticulatum* (Chlorophyta) microalgal extracts showed various antioxidant activities using different assays [66]. Moaveni et al. [67] reported, for the first time, that *Schizochytrium limacinum* (Thraustochytriaceae, Labyrinthulomycetes) could demonstrate a potential source of bioactive peptides with antioxidant activities. Therefore, *S. limacinum* demonstrates interesting properties for nutraceutical production or is used directly as a dietary intervention to prevent diseases associated with free radicals.

In the same context, seaweeds contain various inorganic and organic substances that can benefit human health [68]. Marine macroalgae gained their importance because they can synthesize a wide variation of secondary metabolites and also as a source of bioactive compounds, such as sulfated polysaccharides, proteins, amino acids, peptides, lipids, minerals, sterols, pigments, and some vitamins. Thus, seaweeds are recognized as renewable good antioxidant resources due to their capacity to ameliorate the oxidative damage caused by ROS and its negative effect [68].

**Figure 5:**
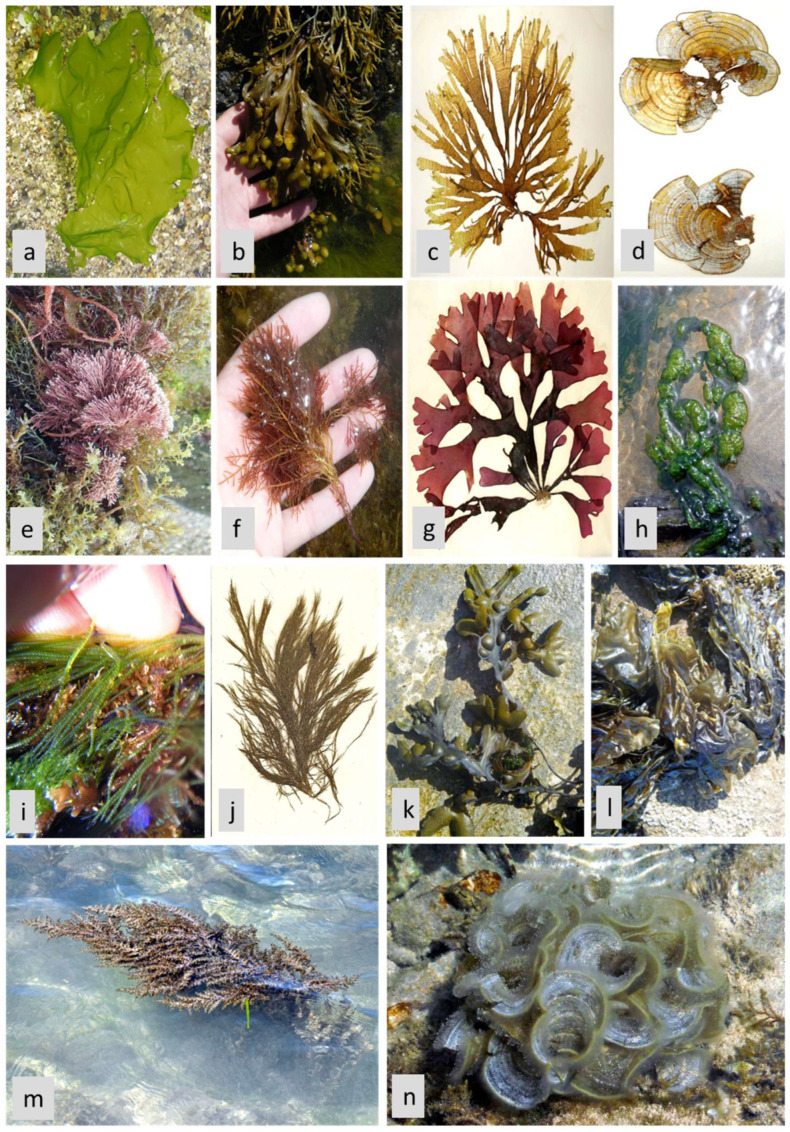
Seaweed species images: (**a**)—*Ulva lactuca* (C); (**b**)—*Fucus spiralis* (P); (**c**)—*Taonia atomaria* (P); (**d**)—*Padina pavonica* (P); (**e**)—*Jania rubens* (R); (**f**)—*Pterocladiella capillacea* (R); (**g**)—*Chondrus crispus* (R); (**h**)—*Ulva intestinalis* (P); (**i**)—*Chaetomorpha aerea* C); (**j**)—*Cladophora rupestris* (C); (**k**)—*Fucus vesiculosus* (P); (**l**)—*Porphyra* (R); (**m**)—*Polycladia myrica* (P); (**n**)—*Padina boergesenii* (P); (C)—Chlorophyta; (R)—Rhodophyta; (P)—Phaeophyceae.

Furthermore, seaweeds possess high concentrations of phenolic molecules, making them one of the greatest sources of natural antioxidants [69]. Several hundred molecules are classified as phenolic compounds because they have a benzene ring structure attached to at least one hydroxyl group [70]. Phenolic compounds can function as antioxidants by chelating the metal ions, such as Cu and Fe, which catalyze free radical generation reactions, and by enhancing the endogenous antioxidant system [71]. With this connection in mind, many studies investigated the antioxidant activity of different macroalgae extracts. The methanolic extract of *Centroceras clavulatum* (Rhodophyta) exhibited high antioxidant activity with various tested assays [70]. In vitro antioxidant assays including DPPH, ABTS (2,2’-azino-bis(3-ethylbenzothiazoline-6-sulfonic acid), and FRAP (ferric reducing antioxidant power) of the methanol extract from *Ulva lactuca* (Chlorophyta) (Figure 5a), showed an outstanding ROS-scavenging potential [72].

Furthermore, Unnikrishnan et al. [28] concluded the antioxidant activity of the ethyl acetate extract of *Sargassum polycystum* and the acetone extract of *Sargassum wightii* (Ochrophyta, Phaeophyceae). In addition, the antioxidant analysis showed that protein hydrolysate-ultrafiltrate fractions FSPH-UF derived from *Fucus spiralis* (Ochrophyta, Phaeophyceae) (Figure 5b) exhibited a significantly higher scavenging of DPPH radical and ferrous ion-chelating (FIC) activity assay, besides a higher FRAP. This activity was due to the bioactive peptides and polyphenols released during enzymatic hydrolysis [73].

Results from the study by Vijayan et al. [74] clearly demonstrate that *S. wightii* fraction obtained by *S. wightii* ethyl acetate extraction showed potent antioxidant activity using DPPH, ABTS, and FRAP assays. Also, the methanolic extract of the brown algae *Turbinaria decurrens* contained a high concentration of polyphenols and exhibited a broad spectrum of antioxidant activity by showing potent radical scavenging activity using ABTS and DPPH free radicals assays, as well as a high ability to reduce copper ions [75].

Moreover, in the study by Ismail et al. [58], the authors showed that the acetone extract of *T. decurrens* showed the highest antioxidant effect of the tested extracts by using DPPH, reducing power, and total antioxidant capacity assays. In addition, the ethanol extract of *Taonia atomaria* brown algae (Figure 5c) recorded the highest antioxidant potential based on various tested assays El-Sheekh et al. [76]. The methanolic extract of *Padina pavonica* (Ochrophyta, Phaeophyceae) (Figure 5d) established the highest DPPH radical scavenging activity of 55.7% ± 0.1 at 50 μg/mL [77].

Likewise, the results of the investigation of Abhishek et al. [78] demonstrated that methanol solvent was successful in extracting polyphenols from *Padina boryana* (Ochrophyta, Phaeophyceae), which was strongly correlated with its antioxidant activity. The enzymatically degraded polysaccharide from *Sargassum fusiforme* brown algae possessed superior antioxidant activity on scavenging HO˙, O_2_^⋅−^ and DPPH˙ radicals [79]. Additionally, the freeze-dried samples of the brown alga *P. pavonica* extracted with ethanol had superior antioxidant activity in hydrogen atom transfer assays of DPPH and ORAC, and with the electron transfer assays of FRAP [80].

Additionally, the ethyl acetate fraction from the red seaweed *Laurencia dendroidea* had the highest antioxidant activity when evaluated by DPPH radical scavenging assay, with a recorded IC_50_ value of 312.09 μg/mL [81]. Moreover, the results of El Nur et al. [82] indicated that the ethanol crude extract of *Jania rubens* (Rhodophyta) (Figure 5e) exhibited the most potent antioxidant activity of 86% using DPPH assay. Likewise, the methanolic extract of *Pterocladiella capillacea* red alga (Figure 5f) possessed a large amount of total polyphenols, which was responsible for its elevated antioxidant potential [83]. Moreover, the methanolic extract of *Chondrus crispus* (Rhodophyta) (Figure 5g), obtained from the Red Sea, was found to contain several flavonoids, polyphenols, and tannins compounds, which displayed remarkable antioxidant activity [84]. Similarly, Murugesan et al. [85] mentioned that the methanol extract of the red seaweed *Ahnfeltiopsis pygmaea* (formerly *Gymnogongrus pygmaeus*) was a valuable source of antioxidants to cure oxidative-stress triggered diseases.

### 3.4. The Inhibitory Activity of Different Algal Extracts on the Carbohydrate Hydrolyzing Enzymes

Regular consumption of functional foods appears to be associated with improved antioxidant enzymes and the suppressed production of pro-inflammatory cytokines, insulin sensitivity, and hypocholesterolemia functions, which are considered essential to preventing and controlling diabetes mellitus. There have been indications that microalgae could be used as antidiabetic foods/ingredients (as shown in Table 1), but the mechanisms of action remain unclear [86,87].

Gouda et al. [61] reported the inhibition of α-glucosidase activity by *Spirulina* butanol extract with an IC_50_ of 23 μg/mL. The data of Priatni et al. [88] showed that the highest inhibition of α-glucosidase activity was 14.02%, recorded for *Pseudanabaena* sp. (Cyanobacteria) exopolysaccharides extract compared to the other studied microalgae. The aqueous and methanolic extracts of *Euglena cantabrica* (Euglenozoa) microalgae displayed the highest antioxidant activity due to the presence of high phenolics content on these extracts [89]. Results of the study by Ahmed et al. [90] revealed that aqueous extracts of *Fischerella* BS1-EG (Cyanobacteria) demonstrated potential inhibition activity for α-glucosidase of 7.5%, indicating its antidiabetic effect. Also, fucoxanthin extracted from *Phaeodactylum tricornutum* (Bacillariophyta) showed strong inhibitory activity toward α-amylase in a concentration-dependent manner, with an IC_50_ value of 0.68 mmol/L and inhibitory activity against α-glucosidase, with an IC_50_ value of 4.75 mmol/L [91]. Likewise, the ethyl acetate extract of *Nannochloropsis oculata* exhibited the highest level of α-amylase inhibition of 78.52% at a maximum concentration of 1000 μg/mL, with an IC_50_ value of 121.96 μg/mL. The same extract exhibited a significant inhibitory action on the α-glucosidase enzyme by 80.42% at the concentration of 1000 μg/mL with an IC_50_ value of 178.53 μg/mL [92].

Recently, Gheda et al. [57] recorded that the methanolic extract of *A. platensis* exhibited the maximum α-amylase enzyme inhibition activity of 96.46% with an IC_50_ value of 13.31 mg/mL compared to the pharmaceutical drug acarbose recorded 1.59 mg/mL. The same extract was also having a strong α-glucosidase inhibitory activity of 97.42% and an IC_50_ value of 9.56 mg/mL compared to acarbose’s IC_50_ value of 1.03 mg/mL. Moreover, the study by Priatni etal. [93] showed that the methanolic extract of the marine microalgae *Porphyridium* sp. (Rhodophyta) was the strongest among the studied microalgae in inhibiting the α-glucosidase enzyme activity (12.63%).

**Table 1:** The inhibitory activity (%) of microalgal species extracts on the carbohydrate hydrolyzing enzymes *.

Algal Species	Division	Extract Type	α-Amylase Inhibition %	IC_50_ Value	α-Glucosidase Inhibition %	IC_50_ Value	Ref.
*Spirulina* sp.	Cyanobacteria	Butanol crude extract	-	-	-	23 μg/mL	[61]
*Pseudanabaena* sp.	Cyanobacteria	Exopolysaccharides extract	-	-	14.02%	-	[88]
*Fischerella* sp.	Cyanobacteria	Aqueous crude extract	-	-	7.5%	-	[90]
*Phaeodactylum tricornutum*	Bacillariophyta	Fucoxanthin extract.	-	0.68 mmol/L	-	4.75 mmol/L	[91]
*Nannochloropsis oculata*	Ochrophyta	Ethyl acetate crude extract	78.52%	121.96 μg/mL	80.42%	178.53 μg/mL	[92]
*Arthrospira platensis*	Cyanobacteria	Methanol crude extract	96.46%	13.31 mg/mL	97.42%	9.56 mg/mL	[57]
*Porphyridium* sp.	Rhodophyta	Methanol crude extract	-	-	12.63%.	-	[93]

* Values of the inhibitory activity (%) was cited for the maximum concentration of the extract for both enzymes.

Seaweeds contain various inorganic and organic substances that can benefit human health [94]. Due to their ability to synthesize a wide range of secondary metabolites and as a source of bioactive compounds, such as sulfated polysaccharides, proteins, pigments, fatty acids, peptides, lipids, minerals, sterols, and phenolic compounds, seaweeds are known as good renewable sources of antioxidant materials. Furthermore, because seaweeds have a high concentration of phenolic molecules, they are regarded as one of the greatest sources of natural antioxidants. [95,96]. Due to their ability to mitigate the oxidative damage caused by excess ROS and its harmful effect, seaweed extracts were recommended by many studies as candidates for antidiabetic drugs (as shown in Table 2). The study by Reka et al. [25] reported that *Ulva reticulata* (Chlorophyta) ethanol extract showed a maximum α-amylase and α-glucosidase inhibition activity of 89.1% and 79.55%, respectively. Moreover, different green (Chlorophyta) seaweed extracts, such as *Ulva intestinalis* (also known as *Enteromorpha intestinalis*) (Figure 5h), *Chaetomorpha aerea* (Figure 5i), and *Cladophora rupestris* (Figure 5j) proved their activity to manage diabetes by inhibiting the carbohydrate digestive enzymes [97]. Mohapatra et al. [98] reported the antidiabetic properties of the ethyl acetate extract of *U. lactuca* by inhibiting the carbohydrate hydrolyzing enzymes due to their bioactive components’ activity. In addition, the ethyl acetate extract of *S. polycystum* and the acetone extract of *S. wightii* have shown significant abilities in inhibiting α-amylase (IC_50_ 438.5 μg/mL) and α-glucosidase (IC_50_ 289.7 μg/mL), and thus can prevent postprandial hyperglycemia [28]. Also, the methanolic extract of *Spatoglossum asperum* (Ochrophyta) showed a significant α-glucosidase inhibitory activity due to the presence of phytochemicals, such as flavonoids, tannins, and saponins [99].

The methanolic extract of *Hormophysa cuneiformis* (Ochrophyta) was the most active, recording an inhibition activity of 53% for the α-glucosidase enzyme at the highest investigated concentration (1000 μg/mL) with an IC_50_ of 676.9 μg/mL [100]. Additionally, a semi-purified phlorotannin fraction of *Fucus vesiculosus* (Ochrophyta, Phaeophyceae) (Figure 5k) showed potent inhibitory effects against α-amylase and α-glucosidase enzymes with IC_50_ values of 2.8 and 0.82 μg/mL, respectively, compared to the pharmaceutical drug acarbose (IC_50_ = 206.6 μg/mL) [101].

A similar conclusion was reported by Arguelles and Sapin [75], where the methanolic extract of *T. decurrens* brown macroalgae presented a potent inhibitory activity (IC_50_ of 11 μg/mL) for α-glucosidase in vitro, as compared to using acarbose and metformin as antidiabetic drugs. Besides, the acetone extract of *T. decurrens* also showed the highest inhibitory effects for both carbohydrate hydrolyzing enzymes α-amylase (96.1%), with an IC_50_ value of 4.37 mg/mL and α-glucosidase (97.4%), with an IC_50_ value of 2.84 mg/mL, which was attributed to its total phenolic content capability [58]. Among the recommended seaweed extracts, the ethanol extract of *T. atomaria* (Figure 5c) demonstrated a maximum α-amylase inhibition capacity of 66.3% [77].

Moreover, Tessema [102] reported the inhibitory effect of the protein fraction extracted from the red alga *Porphyra* sp. (Figure 5l) on the carbohydrate-related enzymes. The ethyl acetate fraction from the red seaweed *L. dendroidea* showed a strong in vitro α-glucosidase inhibitory activity with an IC_50_ value of 8.14 μg/mL [81]. Similarly, the result of the study by Sanger et al. [103] revealed that various phytochemical constituents detected in the aqueous extract of *Halymenia durvillei* red alga were responsible for its antidiabetic potential via the inhibition of α-glucosidase activity (IC_50_ of 4.34 mg/mL).

**Table 2:** The inhibitory activity (%) of macroalgal species extracts on the carbohydrates hydrolyzing enzymes *.

Algal Species	Division	Extract Type	α-Amylase Inhibition %	IC_50_	α-Glucosidase Inhibition %	IC_50_	Ref.
*Ulva reticulata*	Chlorophyta	Ethanol crude extract	89.1%	-	79.55%	-	[25]
*Sargassum polycystum*	Phaeophyta	Ethyl acetate	-	438.5 μg/mL	-	289.7 μg/mL	[28]
*Sargassum wightii*	Phaeophyta	Acetone crude extract	-	438.5 μg/mL	-	289.7 μg/mL	[28]
*Hormophysa cuneiformis*	Phaeophyta	Methanol crude extract	-	-	53%	676.9 μg/mL	[100]
*Fucus vesiculosus*	Phaeophyta	Semi-purified phlorotannin fraction	-	2.8 μg/mL	-	0.82 μg/mL	[101]
*Turbinaria decurrens*	Phaeophyta	Methanol crude extract	-	-	-	11 μg/mL	[75]
*Turbinaria decurrens*	Phaeophyta	Acetone crude extract	96.1%	4.37 mg/mL	97.4%	2.84 mg/mL	[58]
*Taonia atomaria*	Phaeophyta	Ethanol crude extract	66.3%	-	-	-	[77]
*Laurencia dendroidea*	Rhodophyta	Ethyl acetate fraction	-	-	-	8.14 μg/mL	[81]
*Halymenia durvillei*	Rhodophyta	Aqueous crude extract	-	-	-	4.34 mg/mL	[103]

* Values of the inhibitory activity (%) was cited for the maximum concentration of the extract for both enzymes.

### 3.5. In Vivo Antihyperglycemic Activity of Different Algal Extracts

Administration of cyanobacteria leads to regaining body weight in streptozotocin (STZ)-induced diabetic rats and also reverses the hepatic damage by renormalizing serum hepatic marker enzymes. Cyanobacteria show anti-hypoglycemic action through the potentiation of pancreatic insulin secretion from the intact β-cells of the islets. In a recent study, diabetic rabbits, when fed with *A. platensis* cyanobacterium powder, showed antihyperglycemic activity by lowering the animal’s blood glucose level [104]. Moreover, the administration of 400 mg/kg of *A. platensis* powder could reduce the adverse effect of hyperglycemia in alloxan-induced diabetic rats [105]. Furthermore, the antioxidant compounds detected in *A. platensis* extract can aid in preventing diabetes or alleviate its adverse effects on blood parameters and the inflammatory phase [106]. In a study conducted by Gheda et al. [57], diabetic rats which were given *A. platensis* methanol extract showed a significant reduction in elevated glucose levels, liver functions, renal functions, total bilirubin, and lipid profile. In addition, compared to the diabetic control group of rats, the same extract caused a regain in body weight loss, protein profile, albumin, hemoglobin, and HDL levels. The extract also improved the histological tissue damage due to diabetes induction in the liver and pancreatic tissues of the treated rats without causing any negative effects.

Moreover, the effect of *C. vulgaris* green microalgae as an antioxidant supplement for diabetic rats boosted the recovery effect on their hematological parameters [107]. Also, Kawee-Ai et al. [91] reported the antidiabetic property of *P. tricornutum* fucoxanthin extract. Likewise, Nasirian et al. [108] reported that oral administration of *N. oculata* microalgae was able to reverse the negative effect of STZ by reducing the lipid profile and glucose (except HDL-C), and by increasing insulin and HDL-C in diabetic rats. It also recovered the body weight loss in the diabetic rats. These effects were attributed to some components present in the microalgae powder, such as fibers content, lipid profile, and antioxidant pigments.

Many studies documented the biological properties of green seaweeds. In a complementary study by Mohapatra et al. [109], the authors endorsed the ability of the *U. lactuca* ethyl acetate extract as an additive therapy during diabetes treatment. The extract could reverse the complications related to diabetes and was non-toxic to the important tissues. A promising protective effect of *U. reticulata* aqueous extracts against diabetic complications generated by STZ-mediated oxidative stress was reported in [25,110]. Diabetic rats treated orally with ethanolic extract of the green macroalgae *U. reticulata* for 45 days resulted in a significant reduction in fasting plasma glucose, thiobarbituric acid reactive substances (TBARS), and lipid hydroperoxides. Furthermore, using this extract elevated the activities of plasma insulin, vitamin E, and vitamin C and reduced glutathione (GSH) content when compared with the diabetic control group. A study by Labbaci and Boukortt [111] indicated that consumption of *U. lactuca* seaweed and its hydroethanolic extract mitigated insulin resistance, which plays a fundamental role in the pathogenesis of diabetes and helps regenerate damaged pancreatic β-cells. In addition, *U. lactuca* and its hydroethanolic extract may have anti-atherosclerotic effects by improving reverse cholesterol transport. Such results may have major therapeutic promise for helping to prevent the onset of complications in diabetic patients.

Many researchers reported that most brown algae are rich in important secondary metabolites, which had in vivo antidiabetic activities [112]. For example, Na-alginate from *Turbinaria ornata* exhibited antihyperlipidemic and antidiabetic activities through the reduction of blood glucose and other diabetic-boosted physiological changes [113]. It was reported in recent studies that an aqueous extract of *Padina boergesenii* (Ochrophyta) was found to have a vital effect on the reduction of elevated blood glucose levels, kidney marker levels, and liver function lipid profile, in addition to its hepatoprotective activity [114,115]. Likewise, Pirian et al. [14] determined the antidiabetic and antioxidant potential of the methanolic extracts for both *Polycladia myrica* (Figure 5m) and *Sirophysalis trinodis* (Ochrophyta, Phaeophyceae). In addition, the preprandial administration of the brown alga *Ascophyllum nodosum* extract was able to control the hyperglycemia of diabetic animals [116]. The same effect was observed for the brown alga *Sargassum hystrix’s* extracts at a dose of 300 mg/kg. This extract was able to lower the levels of preprandial and postprandial glucose levels of STZ-induced diabetic rats and reversed the weight loss of the rats, triglycerides, and cholesterol levels to their normal (control) levels. Moreover, this dose (300 mg/kg) had the best capability to prevent necrosis of the pancreas in diabetic rats [117]. The data of Akbarzadeh et al. [118] mentioned that the hydroalcoholic extract of the brown macroalgae *Sargassum oligocystum* at a dose of 300 mg/kg had a healing effect on diabetic induced rats by reducing insulin resistance, decreasing glucose concentration and triglyceride, and regenerating of damaged pancreatic β-cells of the STZ-induced diabetic rats.

Recently, an in vivo investigation conducted by Abdel-Karim et al. [119] revealed that oral administration of rats with *T. decurrens* acetone extract at 300 mg/kg dose exhibited antihyperglycemic activity against alloxan-induced diabetes by reducing the elevated blood glucose level, remarkably decreasing the liver and kidney functions, and reducing the hyperlipidemia related to diabetes. In addition, as compared to untreated diabetic rats, treatment with the same extract resulted in a recovery in body weight loss, total protein, albumin, and hemoglobin levels. Furthermore, treatment of rats with the same extract alleviated diabetes-related liver and pancreatic histopathological abnormalities.

Concerning the biological activities of red seaweeds, Murugesan et al. [120] documented the antidiabetic activity of the red alga *Grateloupia lithophila* methanolic extract, which could inhibit the diabetic enzymes α-amylase and α-glucosidase. Radhika and Priya [121] revealed that ethanol extract of the red alga *Acanthophora spicifera* was able to reduce blood glucose levels and the hematological and biochemical parameters linked to diabetes and could also improve the loss in body weight. In the same manner, the red alga *Gelidium amansii* as a diet powder could control diabetes of STZ-induced diabetic rats via reducing their plasma glucose level, lipids, adipocytokines, and adipose tissue weight [122]. The efficacy of sulfated galactopyran compound from *Gracilaria opuntia* against diabetes and the histological changes related to it was reported by Rayapu et al. [123]. Also, the aqueous extracts of *Gracilaria edulis* possessed an inhibitory activity on the carbohydrate hydrolyzing enzymes [25]. In vivo results of the study by Nguyen et al. [81] revealed that the ethyl acetate fraction of the red seaweed *L. dendroidea* could significantly suppress the glucose level of alloxan-diabetic mice, and the oral administration of the same extract was not toxic at a dose of 100 mg/kg of body weight, as determined by body weight changes and liver biochemical parameters.

#### 3.5.1. Effect of Different Algal Extracts as Treatment on the Blood Glucose Levels of Diabetic-Induced Animals

In a recent study [104], diabetic rabbits were fed with *A. platensis* powder, which showed antihyperglycemic activity by lowering their blood glucose levels. Similarly, the administration of *A. platensis* powder (400 mg/kg) could reduce the adverse effect of hyperglycemia in alloxan-induced diabetic rats [105]. El-Baz et al. [124] informed that the possible mechanism by which *Arthrospira* (*Spirulina)* brings its antihyperglycemic action may be through improving the pancreatic secretion of insulin from the β-cell islet or due to enhancing the transportation of blood glucose to the peripheral tissue. This was clearly demonstrated, according to the former study results, by the increased levels of insulin in the diabetic rats treated with *Arthrospira*.

Through the same behavior, the increase in postprandial blood glucose level was significantly suppressed by *T. decurrens* acetone extract administration (300 mg/kg body weight) in diabetic rats. These findings suggested that *T. decurrens* acetone extract might slow the absorption of dietary carbohydrates, hence preventing the rise in postprandial blood glucose levels. In a former study, extracts of some brown algae species exposed a beneficial effect in controlling postprandial glucose levels in diabetic obese rats [125].

Likewise, *Sargassum ringgoldianum* methanolic extract [33] and *P. boergesenii* (Ochrophyta, Phaeophyceae) aqueous extract [114] demonstrated a decrease in blood glucose levels in STZ-induced diabetic mice. Likewise, the therapy with brown seaweeds *Sargassum longiotom* “Selvaraj & Palanisamy” ethanolic extract [126] and with *Hydroclathrus clathratus* aqueous extract [127] showed anti-hyperglycemic activity in alloxan-induced diabetic rats. Furthermore, *T. ornata* extract had a strong effect in lowering blood glucose levels in alloxan-induced diabetic rats, as established by the study of Husni et al. [113]. In addition, the oral treatment of diabetic rats with ethyl acetate extracts of *S. wightii* and *U. lactuca* showed remarkable effectiveness in lowering elevated glucose levels [109]. In this regard, one putative mechanism by which such extracts exert an anti-hyperglycemic effect in diabetic rats is by increasing glucose transport across cell membranes and boosting glycogen formation or by enhancing the glycolysis pathway via releasing insulin from degranulation in pancreatic β-cells [114]. Also, these extracts may have insulin-like effects on peripheral tissues, either by promoting glucose absorption, lowering glucose uptake in the gut, and/or blocking hepatic gluconeogenesis [121].

#### 3.5.2. Effect of Different Algal Extracts as Treatment on the Body Weight of Diabetic-Induced Animals

Alloxan-induced diabetes is accompanied by gradual body weight loss, which might be owing to increased muscle wasting or protein breakdown in the tissues [128]. The rats’ diabetes state is frequently associated with a drop in body weight.

Layam and Reddy [129] informed that oral treatment of diabetic rats with *Spirulina* powder at different doses resulted in an increment in their body weights, and many studies reported similar findings. Pandey et al. [130] stated that diabetic rats treated with *Limnospira maxima* (formerly *Spirulina maxima*) showed a regain in their body weight, which may be well explained by either increased insulin secretion or increased food consumption. Likewise, oral administration of *A. platensis* aqueous extract to diabetic rats for 50 days led to an obvious restoring of their body weight, suggesting that this extract substantially improved the general health status and metabolic mechanisms by effective controlling or reversing gluconeogenesis [131]. In addition, Hussaini et al. [105] reported that administration of *A. platensis* powder at 400 mg/kg could significantly reduce the adverse effect of body weight loss in alloxan-inducted diabetic rats after witnessing a significant enhancement in body weight compared to diabetic control rats. Similarly, diabetic rabbits fed with *A. platensis* powder showed a noteworthy regain in their body weight loss [104]. Also, Gheda et al. [57] informed that a significant increment in the body weight of alloxan-induced diabetic rats was recovered by taking different oral treatment doses of *A. platensis* methanol extract compared to the diabetic control (untreated) rats.

In the same manner, Nagy [127] demonstrated the ability of *H. clathratus* (brown seaweed) aqueous extract to restore body weight loss caused by diabetes induction in mice. The ethanol extract from different seaweeds, *A. spicifera* (Rhodophyta), *Caulerpa scalpelliformis* (Chlorophyta), and *Padina tetrastomatica* (Phaeophyceae), improved the loss of body weight of the experimental diabetic rats [121]. Moreover, the oral therapeutic ethyl acetate extracts of *S. wightii* and *U. lactuca* demonstrated considerable activity in improving body weight loss in diabetic rats [109]. Likewise, Abdel-Karim et al. [64] found that administration of *T. decurrens* acetone extract or standard Diabenor drug tended to reverse the loss of body weight due to diabetic-induced effects; meanwhile, the untreated diabetic rats had a significant decrease in body weight.

#### 3.5.3. Effect of Different Algal Extracts as Treatment on the Hemoglobin (Hb) Levels of Diabetic-Induced Animals

Many studies verified that the hemoglobin level is affected by the presence of glucose in the blood. The improvement in the level of Hb in animals supplemented by different doses of *Arthrospira* might be due to the decreased level of blood glucose that automatically led to decreased Hb values [129]. Another reason is that *Arthrospira*, which is a respectable source of iron, might contribute to raising the Hb levels. In a recent study, diabetic rabbits administrated with *A. platensis* powder showed increasing Hb levels [104]. According to Gheda et al. [57], the Hb level was considerably lower than normal following alloxan-induced diabetes in the experimental rats. A substantial increase in Hb levels was recorded in the diabetic rats after treatment with various doses of *A. platensis* extract.

In the same context, Banu and Mageswari [132] demonstrated a therapeutic impact of *U. reticulata* green seaweed in compensating for iron deficiency and boosting Hb levels. The treatment with ethanolic extract of *Turbinaria conoides* and methanolic extracts of *S. wightii* and *T. conoides* resulted in rising Hb levels, which was related to lowering blood glucose levels [123]. Furthermore, treatment with *P. boergesenii* aqueous extract significantly increased Hb levels in diabetic rats [115]. Likewise, another study suggested that the increase in Hb levels might be due to the enhanced glycemic control along with the decreased blood glucose level brought by the treatment with *T. decurrens* extract, which in turn, was directly proportional to glycosylated Hb level [119]. Thus, it was reported that seaweeds might aid in improving iron status through simple absorption by the body, which facilitates the control of the Hb level in the blood. In contrast to these reports, Radhika and Priya [121] discovered that after inducing diabetes in rats with alloxan, the blood Hb level increased, and treating these diabetic rats with seaweeds could lower it.

#### 3.5.4. Effect of Different Algal Extracts as Treatment on the Total Bilirubin of Diabetic-induced Animals

Bilirubin concentrations may reflect the state of the liver and the type of damage it has received [133]. According to Dey et al. [134], the improvement in hepatic function was caused by lowering levels of free fatty acids and associated peroxides in the blood, as well as lowering levels of oxidation and hepatic inflammation. The possibility of restoring liver-execratory functions in diabetic rats was established due to the administration of *Aphanizomenon flos-aqua* (Cyanobacteria) ethanolic extract and insulin-like protein [135]. Bilirubin levels were expressively amplified after prompting diabetes with alloxan in rats [57]. The total bilirubin value of the diabetic rats treated with different doses of *A. platensis* methanolic extract exhibited a significant reduction. Similarly, the total bilirubin level in diabetic control rats was diminished following oral administration of various dosages of *T. decurrens* extract, as reported by Abdel-Karim et al. [119].

#### 3.5.5. Effect of Different Algal Extracts as Treatment on the Liver Enzymes of Diabetic-Induced Animals

The levels of aminotransferase enzymes, aspartate aminotransferase (AST), and alanine aminotransferase (ALT) in blood serum [136] are a primarily valuable aid in the diagnosis of liver disease as markers of liver toxicity [137] and also reflect hepatocellular necrosis [138]. In earlier studies, the increment of aminotransferase enzyme activities under deficiency of insulin was reported to be responsible for the increased ketogenesis and gluconeogenesis during diabetic disorders [139]. Changes in serum enzymes in diabetic rats were closely connected with changes in the metabolic function of both AST and ALT enzymes. [140]. The mechanism by which blood levels of both aminotransferases were elevated in diabetic rats may involve the increased release of these enzymes from organs, primarily the liver, as a result of oxidative stress or the production of progressive glycosylation end products, as well as liver dysfunction [137]. As reported by Ohaeri [141], liver tissue is necrotized in induced diabetic rats. As a result, an increase in ALT and AST activity in the serum might be due to the escaping of these enzymes from the cytosol of the liver into the bloodstream, indicating a hepatotoxic impact in the diabetic rats. As a diabetic inducer, alloxan injection was toxic and had a detrimental effect on hepatic tissues followed by an increase in AST and ALT enzymes content [57,131]. As recommended by Panigrahi et al. [142], *A. platensis* extract exhibited anti-inflammatory, antioxidant, membrane-stabilizing, and immune-correcting actions, and thus boosted hepatoprotective properties. These outcomes were in agreement with those mentioned by El-Baz et al. [124], who reported that *A. platensis* ethanolic extract could reduce AST and ALT levels. Also, Salem et al. [143] investigated the therapeutic effect of *A. platensis* powder and informed the notable reduction of hepatic enzyme activities.

Similarly, dietary supplementation of diabetic rats with *A. platensis* powder showed a significant beneficial effect in reducing serum hepatic AST and ALT compared with the induced diabetic rats [108,144]. In addition, Ripa et al. [104] documented the potent activity of *A. platensis* powder for the reduction of raised hepatic enzymes levels of diabetic rabbits. After administration of *A. platensis* extract as treatment, a decrease of the serum ALT and AST activities might trigger subsequent alleviation of the liver damage. The treatment with 15 mg/kg body weight dose of *A. platensis* extract was significantly effective in reduction of hepatic transaminase activities compared to the alloxan-diabetic control rats [57].

Furthermore, some studies investigated the effect of macroalgae as reducing agents for hepatic enzymes. Selvaraj and Kumar [126] suggested that ethanolic extract of *S. longiotom* “Selvaraj & Palanisamy” *nom. Inval.* may prevent hepatic injury associated with diabetes. The extract could reduce the levels of serum glutamic-oxaloacetic transaminase (SGOT) and serum glutamic pyruvic transaminase (SGPT). These enzymes are enzyme markers that reflect the necrosis of the hepatocellular by liberating into the blood stream after damaging the cell membrane. Also, oral administration of *H. clathratus* aqueous extract resulted in a significant reduction in hepatic enzyme levels. [127]. According to Dey et al. [134], amelioration of hepatic transaminases resulted from the reduction of free fatty acids content and their peroxides in the blood serum, as well as reduction of the oxidation and hepatic inflammation. Similarly, Abdel-Karim et al. [119] reported that alloxan-diabetic rats treated with *T. decurrens* acetone extract could considerably compact the two parameters of ALT and AST levels in the blood serum, indicating liver function recovery.

#### 3.5.6. Effect of Different algal Extracts as Treatment on the Urea and Creatinine of Diabetic-Induced Animals

The most often used test for screening renal functions is the determination of blood urea. When combined with creatinine readings, urea levels can help in the differentiation of three kinds of azotemia (abnormally high levels of nitrogen-containing compounds in the blood). As the increased creatinine and urea levels represented a decrease in the glomerular filtration rate, the alloxan-induced diabetic rats demonstrated renal impairment. Thus, according to Kumar et al. [114], this was the leading cause of end-stage renal failure that necessitated dialysis or a kidney transplant.

It has been reported that alloxan caused a considerable increase in serum urea and creatinine. In kidney tissue, alloxan increased the production of reactive oxygen species, increased protein carbonylation, and lipid peroxidation, and lowered intracellular antioxidant defense [145]. As suggested by Khan et al. [146], *A. platensis* extract exhibited a nephron-protective effect against diabetic-induced nephropathy. Likewise, Avdagić et al. [147] reported that *Arthrospira* could decrease lipid peroxidation and elevate antioxidant levels, thus considerably modifying renal damage. El-Baz et al. [124] reported the potent activity of *A. platensis* ethanolic extract in reducing urea and creatinine levels to reach 31.00 and 0.96 mg/dl, respectively, when administrated to diabetic-induced rats. Also, Abbas et al. [144] reported the good effect of *Arthrospira* on the decrement of urea and creatinine levels. As explained by Ripa et al. [104], this mitigation effect may be due to the potential antioxidant properties of *A. platensis* extract that improved the renal function via attenuation of the oxidative stress-mediated decline in kidney function. The same observations were informed by Gheda et al. [57], where the elevated levels of urea and creatinine were significantly reduced to the standard levels in the alloxan-induced diabetic rats after treatment with different dosages of *A. platensis* methanolic extract.

Nagy [127] reported a significant reduction in urea and creatinine serum values after oral administration of *H. clathratus* aqueous extract. Also, the water extract of *P. boergesenii* brown seaweed could efficiently lower the elevated levels of urea, uric acid, and creatinine in diabetic rat serum [115]. According to Abdel-Karim et al. [119], The oral treatment of alloxan diabetic rats with *T. decurrens* extract had a considerable decreasing impact on blood urea and creatinine levels, notably at the dose of 300 mg/kg of body weight.

#### 3.5.7. Effect of Different Algal Extracts as Treatment on the Total Protein of Diabetic-Induced Animals

The total protein concentration and percentage indicated by separate fractions may deviate significantly from normal values during disease [148]. Total protein measurements aides in the diagnosis and treatment of diseases involving the kidney, liver, bone marrow, and other nutritional or metabolic problems [149].

The reduction of total protein content linked to diabetes may be a result of the decrease in the three major phases of protein secretion, intracellular transport and discharge, and/or due to increasing protein excretion [150]. The role of microalgae as biomass or extract to combat the rise in the protein content linked to diabetes has been proven by different researchers. For instance, the improvement of protein levels in the blood serum of the diabetic rats treated with *A. platensis* ethanolic extract at a dose of 15 mg/kg body weight was informed by Senthilkumar and John [151]. *A. platensis* ethanolic extract at a dose of 15 mg/kg of body weight was reported to improve the decreased protein level related to STZ-induction in the diabetic rats [124]. Also, Salem et al. [143] mentioned that *A. platensis* powder at 15 mg/kg body weight dose was able to restore the reduced total protein levels as a reverse of diabetes effects. The same enhancement of the protein profile has been observed in the alloxan-induced diabetic rats treated with 15 mg/kg of *A. platensis* methanolic extract [57]. This improvement was ascribed to the immuno-stimulatory effect and the antioxidant property of *A. platensis,* besides its role in improving the hepatic function and/or its richness of proteins, as explained by Venkataraman [152]. Furthermore, *A. platensis* is composed of numerous amino acids, so it may have become a direct source of protein for mice and produced several beneficial metabolic effects [153].

In the same direction, seaweeds were indorsed with the ability to mitigate the elevation in the total protein content related to diabetes disorders. The upgrading of total protein may be attributable to the marked change in the circulating amino acids level, hepatic amino acids uptake, and muscle output of different amino acid concentrations [154]. This effect may also be due to the improvement in either albumin or globulin content or both [155]. Similar outcomes were conveyed by Kumar et al. [114] and Kumar et al. [115], who informed notable improvement in the protein profile after treating diabetic rats with the aqueous extract of *P. boergesenii* brown seaweed. Also, Abdel-Raouf et al. [156] reported the potent effect of the ethanolic extract of *H. cuneiformis* brown alga in the promotion of total protein levels. The decrement of total protein in the induced diabetic rats was significantly improved upon the treatment with *T. decurrens* acetone extract at a dosage of 300 mg/kg of body weight, as mentioned by Abdel-Karim et al. [119].

#### 3.5.8. Effect of Different Algal Extracts as Treatment on the Albumin Level of Diabetic-Induced Animals

Hypoalbuminemia is a prevalent condition in diabetic animals that is usually linked to a decrease in total protein content [155]. Diabetes-related hypoproteinemia can be caused by reduced protein synthesis, increased protein breakdown, and/or increased urine protein excretion [157]. As recommended by several studies, the administration of microalgae and cyanobacteria may elevate the albumin level in the blood serum. It has been reported that the oral intake of *A. platensis* ethanolic extract at 15 mg/kg of body weight could raise the reduced levels of albumin in diabetic rats [124]. Also, Gheda et al. [57] informed of an improvement in the albumin level by treating the diabetic rats with *A. platensis* methanolic extract, though the diabetic control rats exhibited a great reduction in the albumin levels. In the study conducted by Salem et al. [143], the treatment of diabetic rats with *A. platensis* powder (15 mg/kg) exhibited the same improvement effect on the albumin concentration. As previously explained for proteins, the enhancement of albumin levels may be induced due to the antioxidant activity and the stimulated immune effects of different algal extracts, which can improve disturbed liver functions [143]. A significant increase in serum albumin and globulin levels was reported by Abdel-Raouf et al. [156] after the co-administration of diabetic rats with the ethanolic extract of *H. cuneiformis*. In addition, Kumar et al. [115] suggested the ability of *P. boergesenii* to treat the albumin reduction effect resulting from diabetes disorders. The same role was evidenced by Abdel-Karim et al. [119], who detected a significant enhancement in the albumin level of the alloxan-induced diabetic rats following oral administration of *T. decurrens* acetone extract indicating amelioration of the adverse effects caused by diabetes induction.

#### 3.5.9. Effect of Different Algal Extracts as Treatment on the Lipid Profile of Diabetic-Induced Animals

Diabetes frequently involves anomalous lipid metabolism in addition to faulty glucose metabolism, which is regarded as an additional metabolic condition in the diabetic complication series. The activation of lipoprotein lipase and lecithin acyl-cholesterol transferases enhanced the concentration of low-density lipoprotein cholesterol (LDL-C). The elevated levels of very-low-density lipoprotein cholesterol (VLDL-C) and triglycerides (TG) were followed by a decrease in high-density lipoprotein cholesterol (HDL-C) [158]. Normally, insulin induces lipoprotein lipase, which hydrolyzes TG. Insulin shortage leads to a lack of enzyme activation, resulting in hypertriglyceridemia (i.e., increased TG levels in the blood) [159]. Mir et al. [160] established a high content of total lipids in diabetic rabbit blood and ascribed this rise to the enhanced mobilization of free fatty acids from peripheral fat depots. Furthermore, the hyperlipidemia reported in diabetic rats might be explained by insulin insufficiency or the oxidative stress associated with diabetes that can influence lipid metabolism [161]. Salem et al. [143] described that the elevated serum levels of TG, total cholesterol (TC), and LDL were decreased in the diabetic rats given *A. platensis* powder of 15 mg/kg body weight dose and reversed the effect of the reduced HDL level as well. This hypolipidemic activity was possibly triggered by the existence of phenolic compounds in this powder or due to increasing the activity of lipoprotein lipase enzymes in the muscles while decreasing their activity in the adipose tissues [143]. This also indicated that plasma TG was employed for the energy production by the muscle and not for the storage of energy by the adipose tissue [105]. Similar outcomes were recorded by Gheda et al. [57] through the administration of *A. platensis* methanolic extract in diabetic rats, which caused a significant decrease in serum lipid profile level.

Studies also showed that marine algae contain plentiful bioactive constituents that present a potent ability to reduce cholesterol and blood pressure levels, along with encouraging healthy digestion and antioxidant activity [162]. The ethanolic extract of *S. longiotom* “Selvaraj & Palanisamy” *nom. Inval.* exerted a significant beneficial effect on the lipid profile by reducing TG, TC, and LDL levels and significantly increasing the HDL level in the experimental diabetic rats [126]. Oral intake of the aqueous extract of *H. clathratus* caused a significant reduction in either the serum TG levels or TC and LDL-C levels in contrast to a significant elevation in HDL-C [127]. Similarly, treatment with the ethyl acetate extracts of *S. wightii* and *U. lactuca* resulted in the lowering of elevated levels of TG and TC in the diabetes-induced rats [109]. Moreover, the diabetic rats treated with a 400 mg/kg body weight dose of *P. boergesenii* (Figure 5n) aqueous extract reduced the elevated concentrations of TG, TC, and LDL while increasing HDL levels [115]. Also, oral supplementation of diabetic rats with *T. decurrens* extract exhibited a significant positive effect on the lipid profile of the diabetic rats induced by alloxan via a significant reduction of TG, TC, and LDL levels and increased HDL levels [119].

#### 3.5.10. Effect of Different Algal Extracts as Treatment on the Histological Profile of the Liver and Pancreas of the Diabetic-Induced Animals

The liver has a critical role in the excretion and removal of unwanted chemicals from the body. As a result of diabetes induction, characteristic histological changes frequently emerged that show liver disease modifications. The diabetic liver showed hydropic bulging, hepatocyte disarrangement, vacuolization of microvesicles with the removal of nuclei, granular disintegration, and necrosis of liver cells. Also, under diabetes conditions, Zhou et al. [163] and Aboonabi [164] informed severe oxidative damage of the liver tissue. Many studies suggested a valuable role for algae as extract and/or powder form in treating diabetes symptoms, including the hepatic damage effects. The effective role of *A. platensis* ethanol extract (15 mg/kg) was described by [124] to reverse the hepatic histological changes in the rats resulted from diabetes. Abbas et al. [144] reported that the liver of diabetic rats showed apparent normal histological structure after treatment with *Arthrospira* powder at 200 mg/kg of body weight, except for the marked apoptosis of hepatocytes. This recovery effect may be a result of the antioxidant activity of the phenolic compounds present in the *Arthrospira* powder [165,166]. These recommendations were also confirmed by Gheda et al. [57], who showed that the liver of alloxan-induced diabetic rats appeared to be of normal histological structure compared to that of the untreated control rats after treatment with *A. platensis* methanolic extract for 45 days. Therefore, according to studies conducted by El-Baky et al. [167] and Abdel-Daim [168], *Arthrospira* (*Spirulina)* supplementation could act as a potent anti-hepatotoxicity agent.

In the same direction, many studies mentioned the valuable curative role of macroalgal (seaweed) extracts for diabetes and its hepatic complications recovery. The hepatoprotective activity of *S. polycystum* extract against damaged liver tissues was verified by Motshakeri et al. [43]. Likewise, Nagy [127] reported the positive effect of *H. clathratus* aqueous extract against liver injury due to diabetes induction. Treatment with the ethyl acetate extracts of *S. wightii* and *U. lactuca* exhibited a great improvement in the hepatic morphological and physiological disorders caused by diabetes [109]. The necrosis of hepatic cells, changes in microcellular fats, and wide-ranging vacuolization with the vanishing of nuclei were detected in the diabetic rat liver, as observed by Rayapu et al. [123]. These effects were recovered after treatment with different marine algal extracts, viz. *T. conoides* ethanolic extract, *S. wightii* methanolic extract, along with *T. conoides* and *G. opuntia* aqueous extracts.

Kumar et al. [115] reported that *P. boergesenii* aqueous extract had a protective effect on the injured livers of diabetic rats and reduced the exerted histopathological disorders. Similar findings on the recovery effect of *T. decurrens* acetone extract on the diabetic rats’ liver were described by Abdel-Karim et al. [119] after the treatment with a 300 mg/kg body weight dose of this extract.

The pancreas is essential for the control of micronutrient metabolism. As a result, its tissues may be harmed intracellularly during diabetes induction. Due to the lack of visual inspection tools, there is insufficient information on the morphological changes of pancreatic islets with the progression of diabetes [169]. Moreover, any alterations in the systemic metabolism connected to insensitivity, secretion of insulin, and loss of the ability of glycemic control are reflected by alternations in the structure, size, and/or function of the islet [169]. Diabetes has been shown to cause significant histological abnormalities in the characteristic pancreatic Langerhans islets shape, vacuolation of islets, inflammation, and capillary dilatation. This is most visible in diabetic control rats, where there is a reduction in the size of the islets (atrophy), cellular disintegration, and a decrease in the range inhabited by β-cells [57,131].

In the study by Abbas et al. [144], the authors reported that pancreas of diabetic rats treated with *A. platensis* powder showed hyperplasia in the β-cells of the pancreatic tissue and an increased number of Langerhans islets. *A. platensis* supplementation had a potent activity for free radical scavenging and reduced various indicators of toxicity, such as tissue damage in rats [170]. The same recommendation was adopted by Aissaoui et al. [131], who reported the beneficial effect of *A. platensis* powder to reverse the pancreatic damage observed in diabetic animals. In addition, the histopathological investigation of the pancreas revealed that diabetic rats treated with 15 mg/kg of *A. platensis* extract were significantly improved. The histological architecture of the islets appeared with mild vacuolations compared to the diabetic controls [57].

Considering macroalgae, the antidiabetic activity of *H. clathratus* aqueous extract was documented by Mir et al. [160] in addition to its ability to ameliorate the pancreatic damage induced by alloxan. The ethanol extract of *S. polycystum* at 300 mg/kg dose was beneficial in alleviating the histological injuries in the pancreatic tissues in diabetic animals [43]. These observations were also supported by the results of *P. boergesenii* aqueous extract, which displayed a protective effect on pancreatic tissues, allowing for the restoration of the pancreas’ histological deformations [115]. When compared to Diabenor as the standard medicine, treatment of diabetic rats with *T. decurrens* acetone extract at 300 mg/kg dosage had a beneficial impact on the wounded architecture of the pancreas and restored its normal cellular structure [119].

In addition, oral administration of normal rats with different doses of *S. platensis* methanolic extract [57], and *T. decurrens* acetone extract [119] did not display any poisonous effect on the liver or pancreatic tissues when compared to the normal control rats. These observations were well-matched with the results of MTT (3-(4,5-dimethylthiazol-2-yl)-2,5-diphenyltetrazolium bromide tetrazolium) cytotoxicity assay in both studies, which proved the safety of these extracts with no detected differences in rat parameters compared to the normal control ones.

## 4. Conclusions

The diverse anti-diabetic effects of bioactive algal components, which are only applicable to in vitro and in vivo treatments, are covered in this review. Numerous reports have been published on the various functions of the active components that have been extracted from algae. Together, these findings imply that active components derived from algae will be beneficial for the treatment of diabetes. As a result of this, significant anti-diabetic properties of the algal extracts may mark an intriguing advancement in the search for innovative functional uses in numerous industrial uses, including functional foods and pharmaceuticals. Algae-derived compounds proved their potent activity as a natural antioxidant agent and their ability to control postprandial blood glucose levels by inhibiting diabetes-linked enzymes. In addition, the algal extracts could improve the physiological parameters as well as reverse the histological damage related to diabetes mellitus in vivo. The finest possible nutrition and wellness are the goals of algae bioactive components. Despite these high expectations, no product lines have yet demonstrated that they are useful and commercially feasible. Although they are marketed as a functional food and medicine, their market performance falls short of expectations. However, consumer interest in employing natural bioactive compounds as medications has recently increased. Additionally, algae’s numerous biological processes have the potential to boost its value as a health-beneficial ingredient in pharmaceutical and functional food industries.

## Data Availability

Not applicable.

## References

[1] International Diabetes Federation (IDF) (2022). IDF Diabetes Atlas. https://www.diabetesatlas.org/.

[2] WHO (2022). W.H.O. Diabetes. https://www.who.int/news-room/fact-sheets/detail/diabetes.

[3] Ritu M., Nandini J. (2016). Nutritional composition of *Stevia rebaudiana*, a sweet herb, and its hypoglycaemic and hypolipidaemic effect on patients with non-insulin dependent diabetes mellitus. J. Sci. Food Agric..

[4] Akpaso M.I., Igiri A.O., Odey P.A. (2017). A comparative study on the effect of combined methanolic leaf extracts of *Vernonia amygdalina* and *Gongronema latifolium* and metformin on the pancreatic beta cells of streptozocin induced diabetic wistar rats. Asian J. Pharm. Nurs. Med. Sci..

[5] American Diabetes Association (2021). Classification and diagnosis of diabetes: Standards of medical care in diabetes. Diabetes Care.

[6] Armstrong C. (2006). Standards of Medical Care for Patients with Diabetes.

[7] Fox I.S. (2004). Human Physiology.

[8] Vaidyanathan K., Vasudevan D.M., Sreekumari S. (2011). Regulation of blood glucose, insulin and diabetes mellitus. Textbook of Biochemistry for Medical Students.

[9] Xu L., Li Y., Dai Y., Peng J. (2018). Natural products for the treatment of type 2 diabetes mellitus: Pharmacology and mechanisms. Pharmacol. Res..

[10] Franks P.W., McCarthy M.I. (2016). Exposing the exposures responsible for type 2 diabetes and obesity. Science.

[11] Sørensen L.E., Jeppesen P.B., Christiansen C.B., Hermansen K., Gregersen S. (2019). Nordic seaweed and diabetes prevention: Exploratory studies in KK-Ay mice. Nutrients.

[12] Finkel T., Holbrook N.J. (2000). Oxidants, oxidative stress and the biology of ageing. Nature.

[13] Tiwari A. (2001). Imbalance in antioxidant defense and human disease: Multiple approach of natural antioxidant therapy. J. Curr. Sci..

[14] Pirian K., Moein S., Sohrabipour J., Rabiei R., Blomster J. (2017). Antidiabetic and antioxidant activities of brown and red macroalgae from the Persian Gulf. J. Appl. Phycol..

[15] Poljsak B., Fink R. (2014). The protective role of antioxidants in the defence against ROS/RNS-mediated environmental pollution. Oxidative Med. Cell. Longev..

[16] Shin C.S., Moon B.S., Park K.S., Kim S.Y., Park S.J., Chung M.H., Lee H.K. (2001). Serum 8-hydroxy-guanine levels are increased in diabetic patients. Diabetes Care.

[17] Alfadda A.A., Sallam R.M. (2012). Reactive oxygen species in health and disease. J. Biomed. Biotechnol..

[18] Lai M.Y., Fung P.L., Xiaoqiang Y., Zhen-Yu C., Yu H. (2006). Reactive oxygen species in vascular wall. Cardiovasc. Hematol. Disord. -Drug Targets.

[19] Styskal J., Van Remmen H., Richardson A., Salmon A.B. (2012). Oxidative stress and diabetes: What can we learn about insulin resistance from antioxidant mutant mouse models?. Free. Radic. Biol. Med..

[20] Bajaj S., Khan A. (2012). Antioxidants and diabetes. Indian J. Endocrinol. Metab..

[21] Desco M.-C., Asensi M., Márquez R., Martínez-Valls J., Vento M., Pallardó F.V., Sastre J., Viña J. (2002). Xanthine oxidase is involved in free radical production in type 1 diabetes. Diabetes.

[22] Maiese K. (2015). New insights for oxidative stress and diabetes mellitus. Oxidative Med. Cell. Longev..

[23] Xin Y., Yuan B., Yu B., Wang Y., Wu J., Zhou W., Qiu Z. (2015). Tet1-mediated DNA demethylation regulates neuronal cell death induced by oxidative stress. Sci. Rep..

[24] Maiese K. (2015). MTOR: Driving apoptosis and autophagy for neurocardiac complications of diabetes mellitus. World J. Diabetes.

[25] Reka P., Banu T., Seethalakshmi M. (2017). Alpha amylase and α glucosidase inhibition activity of selected edible seaweeds from South Coast area of India. Int. J. Pharm. Pharm. Sci..

[26] Rang H., Dale M., Ritter J., Moore P. (2003). Pharmacology.

[27] Kwon Y.I., Apostolidis E., Kim Y.C., Shetty K. (2007). Health benefits of traditional corn, beans, and pumpkin: In vitro studies for hyperglycemia and hypertension management. J. Med. Food.

[28] Unnikrishnan P.S., Suthindhiran K., Jayasri M.A. (2015). Antidiabetic potential of marine algae by inhibiting key metabolic enzymes. Front. Life Sci..

[29] Bhandari M.R., Jong-Anurakkun N., Hong G., Kawabata J. (2008). α-Glucosidase and α-amylase inhibitory activities of *Nepalese* medicinal herb Pakhanbhed (*Bergenia ciliata*, Haw.). Food Chem..

[30] Fernando M.R., Wickramasinghe S.M.D.N., Thabrew M.I., Ariyananda P.L., Karunanayake E.H. (1991). Effect of *Artocarpus heterophyllus* and *Asteracanthus longifolia* on glucose tolerance in normal human subjects and in maturity-onset diabetic patients. J. Ethnopharmacol..

[31] Lee M.Y., Choi D.S., Lee M.K., Lee H.W., Park T.S., Kim D.M., Chung C.H., Kim D.K., Kim I.J., Jang H.C. (2014). Comparison of acarbose and voglibose in diabetes patients who are inadequately controlled with basal insulin treatment: Randomized, parallel, open-label, active-controlled study. J. Korean Med. Sci..

[32] Kwon Y.I., Apostolidis E., Shetty K. (2008). Inhibitory potential of wine and tea against α-amylase and α-glucosidase for management of hyperglycemia linked to type 2 diabetes. J. Food Biochem..

[33] Lee C.W., Han J.S. (2012). Hypoglycemic effect of *Sargassum ringgoldianum* extract in STZ-induced diabetic mice. Prev. Nutr. Food Sci..

[34] Kalita D., Holm D.G., LaBarbera D.V., Petrash J.M., Jayanty S.S. (2018). Inhibition of α-glucosidase, α-amylase, and aldose reductase by potato polyphenolic compounds. PLoS ONE.

[35] Shibata T., Hama Y., Miyasaki T., Ito M., Nakamura T. (2006). Extracellular secretion of phenolic substances from living brown algae. J. Appl. Phycol..

[36] Desai K., Sivakami S. (2004). *Spirulina*: The wonder food of the 21st Century. Asia-Pac. Biotech News.

[37] Wanasundara P., Shahidi F. (2005). Antioxidants: Science, Technology, and Applications.

[38] Wijesekara I., Senevirathne M., Li Y., Kim S. (2011). Functional ingredients from marine algae as potential antioxidants in the food industry. Handbook of Marine Macroalgae.

[39] Goiris K., De Vreese P., De Cooman L., Muylaert K. (2012). Rapid screening and guided extraction of antioxidants from microalgae using voltammetric methods. J. Agric. Food Chem..

[40] Akoh C., David B.M. (2002). Food Lipids: Chemistry, Nutrition, Biotechnology.

[41] Chandini S.K., Ganesan P., Bhaskar N. (2008). In vitro antioxidant activities of three selected brown seaweeds of India. Food Chem..

[42] Samaraweera A.M., Vidanarachchi J.K., Kurukulasuriya M.S. (2012). Industrial Applications of Macroalgae.

[43] Motshakeri M., Ebrahimi M., Goh Y.M., Matanjun P., Mohamed S. (2013). *Sargassum polycystum* reduces hyperglycaemia, dyslipidaemia and oxidative stress via increasing insulin sensitivity in a rat model of type 2 diabetes. J. Sci. Food Agric..

[44] Gupta V.K.M., Shrivastava R.K., Singh N. (2018). Status of exogenous antioxidant, total antioxidant capacity and oxidative stress in SCA patients. Indian J. Appl. Res..

[45] Abbott I.A., Hollenberg G.J. (1976). Marine Algae of California.

[46] Namikoshi M., Rinehart K.L. (1996). Bioactive compounds produced by cyanobacteria. J. Indian Microbiol..

[47] Raven J.A., Mario G. (2014). Algae. Curr. Biol..

[48] Dhargalkar V.K., Verlecar X.N. (2009). Southern Ocean seaweeds: A resource for exploration in food and drugs. Aquaculture.

[49] Manivannan K., Thirumaran G., Devi G.K., Anantharaman P., Balasubramanian T. (2009). Proximate composition of different group of seaweeds from Vedalai Coastal Waters (Gulf of Mannar). Middle-East J. Sci. Res..

[50] Paul J.P.J. (2013). Phytochemical analysis of *Padina distromatica* Hauck. Indo Am. J. Pharm. Res..

[51] Solanki R., Khanna M., Lal R. (2008). Bioactive compounds from marine actinomycetes. Indian J. Microbiol..

[52] Valls R., Piovetti L., Banaigs B., Archavlis A., Pellegrini M. (1995). (S)-13-hydroxygeranylgeraniol-derived furanoditerpenes from *Bifurcaria bifurcate*. Phytochemistry.

[53] Okwu D.E. (2001). Improving the nutritive value of *Cassava tapioca* meal with local spices. J. Nutraceuticals Funct. Med. Foods.

[54] Rivière C., Hong V.N.T., Pieters L., Dejaegher B., Heyden Y.V., Van M.C., Quetin-Leclercq J. (2009). Polyphenols isolated from antiradical extracts of *Mallotus metcalfianus*. Phytochemistry.

[55] Polterait O. (1997). Antioxidants and free radical scavengers of natural origin. Curr. Org. Chem..

[56] Mandal P., Sinha Babu S.P., Mandal N.C. (2005). Antimicrobial activity of saponins from *Acacia auriculiformis*. Fitoterapia.

[57] Gheda S.F., Abo-Shady A.M., Abdel-Karim O.H., Ismail G.A. (2021). Antioxidant and Antihyperglycemic Activity of *Arthrospira platensis* (*Spirulina platensis*) Methanolic Extract: In vitro and In vivo Study. Egypt. J. Bot..

[58] Ismail G.A., Gheda S.F., Abo-Shady A.M., Abdel-Karim O.H. (2020). In vitro potential activity of some seaweeds as antioxidants and inhibitors of diabetic enzymes. Food Sci. Technol..

[59] Souza M.M.d., Prietto L., Ribeiro A.C., Souza T.D.d., Badiale-Furlong E. (2011). Assessment of the antifungal activity of *Spirulina platensis* phenolic extract against *Aspergillus flavus*. Ciência e Agrotecnologia.

[60] Coulombier N., Jauffrais T., Lebouvier N. (2021). Antioxidant Compounds from Microalgae: A Review. Mar Drugs.

[61] Gouda K.G.M., Kavitha M.D., Sarada R. (2015). Antihyperglycemic, antioxidant and antimicrobial activities of the butanol extract from *Spirulina platensis*. J. Food Biochem..

[62] Jayshree A., Jayashree S., Nallamuthu T. (2016). *Chlorella vulgaris* and *Chlamydomonas reinhardtii*: Effective antioxidant, antibacterial and anticancer mediators. Indian J. Pharm. Sci..

[63] Scaglioni P.T., Quadros L., de Paula M., Furlong V.B., Abreu P.C., Badiale-Furlong E. (2018). Inhibition of enzymatic and oxidative processes by phenolic extracts from *Spirulina* sp. and *Nannochloropsis* sp. Food Technol. Biotechnol..

[64] Abdel-Karim O.H., Gheda S.F., Ismail G.A., Abo-Shady A.M. (2020). Phytochemical screening and antioxidant activity of *Chlorella vulgaris*. Delta J. Basic Appl. Sci..

[65] Santhar D.T., Haq M.A.B., Marudhupandi T., Vaseeharan B., Rajan D.K., Moovendhan M. (2021). Evaluation of chemical compositions and antioxidant potential of marine microalgae of the genus *Nannochloropsis*. Biomass Convers. Biorefinery.

[66] Jaffer M., Ashraf H., Shaheen S. (2021). Phytochemical, antioxidant and antimicrobial activity of biological important algae *Hydrodictyon reticulatum* L. Pak. J. Sci. Ind. Res. Ser. b Biol. Sci..

[67] Moaveni S., Salami M., Khodadadi M., McDougall M., Emam-Djomeh Z. (2022). Investigation of *S.limacinum* microalgae digestibility and production of antioxidant bioactive peptides. LWT.

[68] Zhao C., Yang C., Liu B., Lin L., Sarker S., Nahar L., Yu H., Cao H., Xiao J. (2017). Bioactive compounds from marine macroalgae and their hypoglycemic benefits. Food Sci. Technol..

[69] Gunathilaka T.L., Keertihirathna L.R., Peiris D. (2021). Advanced pharmacological uses of marine algae as an anti-diabetic therapy. Medicinal Plants from Nature.

[70] Elangovan M., Noorjahan A., Anantharaman P. (2019). Extraction Of Metabolites And Screening Their Antioxidant Potential From Marine Macro Algae. Int. J. Sci. Technol. Res..

[71] Al-Azzawie H.F., Alhamdani M.-S.S. (2006). Hypoglycemic and antioxidant effect of *Oleuropein* in alloxan-diabetic rabbits. Life Sci..

[72] Ibrahim R.Y.M., Saber A.A., Hammad H.B.I. (2021). The possible role of the seaweed *Ulva fasciata* on ameliorating hyperthyroidism-associated heart inflammations in a rat model. Environ. Sci. Pollut. Res..

[73] Paiva L., Lima E., Neto A.I., Baptista J. (2017). Angiotensin I-Converting Enzyme (ACE) Inhibitory Activity, Antioxidant Properties, Phenolic Content and Amino Acid Profiles of *Fucus spiralis* L. Protein Hydrolysate Fractions. Mar. Drugs.

[74] Vijayan R., Chitra L., Penislusshiyan S., Palvannan T. (2018). Exploring bioactive fraction of *Sargassum wightii*: In vitro elucidation of Angiotensin 1-converting enzyme inhibition and antioxidant potential. Int. J. Food Prop..

[75] Arguelles E.D., Sapin A.B. (2020). In vitro antioxidant, alpha-glucosidase inhibition and antibacterial properties of *Turbinaria decurrens* Bory (Sargassaceae, Ochrophyta). Asia-Pac. J. Sci. Technol..

[76] El-Sheekh M.M., El-Shenody R.A.E.K., Bases E.A., El Shafay S.M. (2021). Comparative assessment of antioxidant activity and biochemical composition of four seaweeds, Rocky Bay of Abu Qir in Alexandria, Egypt. Food Science and Technology.

[77] Shafay S.E.L., El-Sheekh M., Bases E., El-Shenody R. (2021). Antioxidant, antidiabetic, anti-inflammatory and anticancer potential of some seaweed extracts. Food Sci. Technol..

[78] Abhishek D., Sanjay S., Jadeja B.A. (2021). Cytotoxicity, Antioxidant And Antimicrobial Activity Of Marine Macro Algae *(Iyengaria Stellata* And *Padina Boryana*) From The Gujarat Coast. J. Maharaja Sayajirao Univ. Baroda.

[79] Qian W.-W., Yang S.-Q., Hu S.-M., Wang X.-L., Zhu Y., Zhou T. (2021). Enzymatic degradation, antioxidant and immunoregulatory activities of polysaccharides from brown algae *Sargassum fusiforme*. J. Food Meas. Charact..

[80] Čagalj M., Skroza D., Tabanelli G., Özogul F., Šimat V. (2021). Maximizing the antioxidant capacity of *Padina pavonica* by choosing the right drying and extraction methods. Processes.

[81] Nguyen T.H., Nguyen T.H., Nguyen V.M., Nguyen T.L.P., Tran T.V.A., Do A.D., Kim S.M. (2019). Antidiabetic and antioxidant activities of red seaweed *Laurencia dendroidea*. Asian Pac. J. Trop. Biomed..

[82] El Nur E.E., Ali L.I., Fadul E., Mohamed l.E. (2021). Antioxidant, Antibacterial and Cytotoxic Potential of Selected Macroalgae from the Red Sea, Sudan Coast. Int. Res. J. Biol. Sci..

[83] Hmani I., Ktari L., Ismail A., M’dallel C., El Bour M. (2021). Assessment of the antioxidant and antibacterial properties of red algae (Rhodophyta) from the north coast of Tunisia. Euro-Mediterr. J. Environ. Integr..

[84] Alkhalaf M.I. (2021). Chemical composition, antioxidant, anti-inflammatory and cytotoxic effects of *Chondrus crispus* species of red algae collected from the Red Sea along the shores of Jeddah city. J. King Saud Univ. Sci..

[85] Murugesan S., Vinoth-Kumar R., Kotteswari M., Shanthi N. (2021). In vitro antioxidant activity of marine red alga *Gymnogongrus pygmaeus* J. Agardh. Int. J. Pharm. Res..

[86] Bocanegra A., Macho-Gonzalez A., Garcimartin A., Benedi J., Sanchez-Muniz F.J. (2021). Whole Alga, Algal Extracts, and Compounds as Ingredients of Functional Foods: Composition and Action Mechanism Relationships in the Prevention and Treatment of Type-2 Diabetes Mellitus. Int. J. Mol. Sci..

[87] Ramos-Romero S., Torrella J.R., Pages T., Viscor G., Torres J.L. (2021). Edible Microalgae and Their Bioactive Compounds in the Prevention and Treatment of Metabolic Alterations. Nutrients.

[88] Priatni S., Budiwati T.A., Ratnaningrum D., Kosasih w., Andryani R., Susanti H., Susilaningsih D. (2016). Antidiabetic screening of some Indonesian marine cyanobacteria collection. Bio Diversit AS.

[89] Jerez-Martel I., García-Poza S., Rodríguez-Martel G., Rico M., Afonso-Olivares C., Gómez-Pinchetti J.L. (2017). Phenolic Profile and Antioxidant Activity of Crude Extracts from Microalgae and Cyanobacteria Strains. J. Food Qual..

[90] Ahmed B.E., Badawi M.H., Mostafa S.S., Higazy A.M. (2018). Human Anticancers and Antidiabetic Activities of the Cyanobacterium *Fischerella* sp. BS1-EG Isolated from River Nile, Egypt. Int. J. Curr. Microbiol. Appl. Sci..

[91] Kawee-Ai A., Kim A.T., Kim S.M. (2019). Inhibitory activities of microalgal fucoxanthin against α-amylase, α-glucosidase, and glucose oxidase in 3T3-L1 cells linked to type 2 diabetes. J. Oceanol. Limnol..

[92] Deepa P.K., Subramanian A., Manjusha W.A. (2020). Phytochemical Screening and Evaluation of Antidiabetic Activity of the Marine Microalgae: *Nannochloropsis* sp. Int. J. Life Sci. Pharma Res. (IJLPR).

[93] Priatni S., Ratnaningrum D., Kosasih W. (2021). The Screening of Antidiabetic Activity and The Cultivation Study of Local Marine Microalgae. IOP Conf. Ser. Mater. Sci. Eng..

[94] Rashad S., El-Chaghaby G.A. (2020). Marine Algae in Egypt distribution, phytochemical composition and biological uses as bioactive resources (a review). Egypt. J. Aquat. Biol. Fish..

[95] Satpati G.G., Mal N., Pal R. (2021). Seaweed-based interventions for diabetic complications: An analytical discourse. Syst. Biosci. Eng..

[96] Manach C., Scalbert A., Morand C., Rémésy C., Jiménez L. (2004). Polyphenols: Food sources and bioavailability. Am. J. Clin. Nutr..

[97] Unnikrishnan P.S., Suthindhiran K., Jayasri M.A. (2015). Alpha-amylase inhibition and antioxidant activity of marine green algae and its possible role in diabetes management. Pharmacogn. Mag..

[98] Mohapatra L., Bhattamisra S., Panigrahy R., Parida S. (2016). Evaluation of the antioxidant, hypoglycaemic and antidiabetic activities of some seaweed collected from the East Coast of India. J. Biomed. Pharmacol..

[99] Pandithurai M., Murugesan S., Bhuvaneswari S., Thennarasan S. (2015). In vitro α-amylase and α-glucosidase inhibition activity of methanolic extract of marine brown alga *Spatoglossum asperum*. Int. J. Adv. Pharm..

[100] Osman N.A.H.K., Siam A.A., El-Manawy I.M., Jeon Y.-J. (2019). Anti-microbial and Anti-diabetic Activity of Six Seaweeds Collected from the Red Sea, Egypt. Int. J. Environ. Sci..

[101] Catarino M.D., Silva A.M.S., Mateus N., Cardoso S.M. (2019). Optimization of Phlorotannins Extraction from *Fucus vesiculosus* and Evaluation of Their Potential to Prevent Metabolic Disorders. Mar. Drugs.

[102] Tessema H.A. (2018). Evaluation of the in vitro α-amylase enzyme inhibition potential of commercial dried laver (*Porphyra species*) seaweed protein hydrolysate. Turk. J. Fish. Aquat. Sci..

[103] Sanger G., Rarung L.K., Damongilala L.J., Kaseger B.E., Montolalu L.A.D.Y. (2019). Phytochemical constituents and antidiabetic activity of edible marine red seaweed (*Halymenia durvilae*). IOP Conf. Ser. Earth Environ. Sci..

[104] Ripa S.A., Aziz F.B., Islam R., Hasan M.M., Parvez M.M.M., Lipi T., Jubayar M., Roy M.C. (2018). Antidiabetic effect of *Spirulina* (*Spirulina platensis*) in alloxan induced rabbit model. Int. J. Nat. Soc. Sci..

[105] Hussaini S., Hossain M.I., Islam M.S., Rafiq K. (2018). Effects of *Spirulina platensis* on alloxan induced diabetic rats. Progress. Agric..

[106] Nasirian F., Dadkhah M., Moradi-Kor N., Obeidavi Z. (2018). Effects of *Spirulina platensis* microalgae on antioxidant and anti-inflammatory factors in diabetic rats. Diabetes Metab. Syndr. Obes..

[107] Emami S., Olfati A.O. (2017). Effects of dietary supplementing of *Spirulina platensis* and *Chlorella vulgaris* microalgae on hematologic parameters in streptozotocin-induced diabetic rats. Iran. J. Pediatr. Hematol. Oncol..

[108] Nasirian F., Sarir H., Moradi-Kor N. (2019). Antihyperglycemic and antihyperlipidemic activities of Nannochloropsis oculata microalgae in Streptozotocin-induced diabetic rats. Biomol Concepts.

[109] Mohapatra L., Bhattamisra S., Panigrahy R., Parida S., Pati P. (2016). Antidiabetic effect of *Sargassum wightii* and *Ulva fasciata* in high fat diet and multi low dose streptozotocin induced type 2 diabetic mice. UK J. Pharm. Biosci..

[110] Kumar N., Sharunetha T. (2018). *Ulva reticulata*, a marine alga normalize streptozotocin induced lipid peroxidation in experimental diabetic rats. J. Drug Deliv. Ther..

[111] Labbaci F.Z., Boukortt F.O. (2020). Beneficial Effects of Algerian Green Alga *Ulva lactuca* and Its Hydroethanolic Extract on Insulin Resistance and Cholesterol Reverse Transport in High-Fat/Streptozotocin Diabetic Rats. Prev. Nutr. Food Sci..

[112] Gunathilaka T.L., Samarakoon K., Ranasinghe P., Peiris L.D.C. (2020). Antidiabetic Potential of Marine Brown Algae-a Mini Review. J. Diabetes Res..

[113] Husni A., Pawestri S., Isnansetyo A. (2016). Blood glucose level and lipid profile of alloxan–induced diabetic rats treated with Na-alginate from seaweed *Turbinaria ornata* (Turner) J.agardh. J. Teknol. (Sci. Eng.).

[114] Kumar P.S., Sudha S., Prakash S. (2014). Antidiabetic activity of aqueous extract of *Padina boergesenii* in streptozotocin-induced diabetic rats. Int. J. Pharm. Pharm. Sci..

[115] Kumar R.V. (2017). Antidiabetic Potential of Marine Red Alga *Champia Parvula* (C. Agardh) by Inhibiting Key Metabolic Enzymes. World J. Pharm. Res..

[116] Gabbia D., Carrara M., Martin S.D. (2018). The brown alga *Ascophyllum nodosum* as a nutraceutical useful for the control of type II diabetes. Curr. Res. Diabetes Obes. J..

[117] Gotama T.L., Husni A. (2018). Antidiabetic Activity of *Sargassum hystrix* Extracts in Streptozotocin-Induced Diabetic Rats. Prev. Nutr. Food Sci..

[118] Akbarzadeh S., Gholampour H., Farzadinia P., Daneshi A., Ramavandi B., Moazzeni A., Keshavarz M., Bargahi A. (2018). Anti-diabetic effects of *Sargassum oligocystum* on Streptozotocin-induced diabetic rat. Iran J. Basic Med. Sci..

[119] Abdel-Karim O.H., Abo-Shady A.M., Ismail G.A., Gheda S.F. (2021). Potential effect of *Turbinaria decurrens* acetone extract on the biochemical and histological parameters on alloxan-induced diabetic rats. Int. J. Environ. Health Res..

[120] Murugesan S., Anand Babu M., Bhuvaneswari S., Kotteswari M., Thennarasan S. (2015). In vitro antidiabetic activity of methanolic extracts of selected marine algae. Eur. J. Pharm. Med. Res..

[121] Radhika D., Priya R. (2015). Assessment of antidiabetic activity of some selected seaweeds. Eur. J. Biomed. Pharm. Sci..

[122] Yang T.H., Yao H.T., Chiang M.T. (2015). Red algae (*Gelidium amansii*) reduces adiposity via activation of lipolysis in rats with diabetes induced by streptozotocin-nicotinamide. J. Food Drug Anal..

[123] Rayapu L., Makkar F., Anandan S.K., Maneesh A., Chakraborty K., Valluru L. (2017). Protective role of marine macroalgae extracts against STZ induced diabetic rats. J. Coast. Life Med..

[124] El-Baz F.K., Aly H.F., El-Sayed A.B., Amal A.M. (2013). Role of *Spirulina Platensis* in the control of glycemia in DM2 rats. Int. J. Sci. Eng. Res..

[125] Nam J., Lee W., Yoon I., Kang M., Jang H., Youn J., Kim B., Kong H., Kim K.-H., Kim Y.-h. (2007). Effect of a Brown Algae Extract on Postprandial Glucose Control in Neonatal Diabetic and Obese Rats. FASEB J..

[126] Selvaraj S., Palanisamy S. (2018). Investigations on the anti-diabetic potential of novel marine seaweed *Sargassum longiotom* against alloxan-induced diabetes mellitus: A pilot study. Bangladesh J. Pharmacol..

[127] Nagy M.A. (2015). Biochemical and histopathological analysis of *Hydroclathrus clathratus* aqueous extract on alloxan induced diabetic rats. BioChem. Indian J..

[128] Chatterjea M.N., Shinde R. (2002). Textbook of Medical Biochemistry.

[129] Layam A., Reddy C.L. (2007). Antidiabetic property of S*pirulina*. Diabetol. Croat..

[130] Pandey J., Tiwari A., Mishra G., Mishra R. (2011). Role of *Spirulina maxima* in the control of blood glucose levels and body weight in streptozotocin induced diabetic male Wistar rats. J. Algal Biomass Util..

[131] Aissaoui O., Amiali M., Bouzid N., Belkacemi K., Bitam A. (2017). Effect of *Spirulina platensis* ingestion on the abnormal biochemical and oxidative stress parameters in the pancreas and liver of alloxan-induced diabetic rats. Pharm. Biol..

[132] Banu A.T., Mageswari S. (2015). Nutritional status and effect of seaweed chocolate on anemic adolescent girls. Food Sci. Hum. Wellness.

[133] Yakubu M., Akanji M., Oladiji A. (2005). Aphrodisiac potentials of the aqueous extract of *Fadogia agrestis* (Schweinf. Ex Hiern) stem in male albino rats. Asian J. Androl..

[134] Dey P., Saha M., Chowdhuri S., Sen A., Sarkar M., Haldar B., Chaudhuri T. (2015). Assessment of anti-diabetic activity of an ethnopharmacological plant *Nerium oleander* through alloxan induced diabetes in mice. J. Ethnopharmacol..

[135] Kuriakose G., Kurup M. (2010). Hepatoprotective effect of *Spirulina lonar* on paracetamol induced liver damage in rats. Asian J. Exp. Biol. Sci..

[136] Huang X.J., Choi Y.K., Im H.S., Yarimaga O., Yoon E., Kim H.S. (2006). Aspartate Aminotransferase (AST/GOT) and Alanine Aminotransferase (ALT/GPT) Detection Techniques. Sensors.

[137] Mori D., Baviera A., de Oliveira Ramalho L., Vendramini R., Brunetti I., Pepato M. (2003). Temporal response pattern of biochemical analytes in experimental diabetes. Biotechnol. Appl. Biochem..

[138] Setorki M., Asgary S., Eidi A., Rohani A., Khazaei M. (2010). Acute effects of vinegar intake on some biochemical risk factors of atherosclerosis in hypercholesterolemic rabbits. Lipids Health Dis..

[139] Felig P., Marliss E., Ohman J., Cahill C. (1970). Plasma amino acid levels in diabetic ketoacidosis. Diabetes.

[140] Asayama K., Nakane T., Uchida N., Hayashibe H., Dobashi K., Nakazawa S. (1994). Serum antioxidant status in streptozotocin-induced diabetic rat. Horm. Metab. Res. Horm. Und Stoffwechs. Horm. Et Metab..

[141] Ohaeri O. (2001). Effect of garlic oil on the levels of various enzymes in the serum and tissue of streptozotocin diabetic rats. Biosci. Rep..

[142] Panigrahi B.B., Panda P.K., Patro V.J. (2010). Comparative hepatoprotective activity of different extracts of *spirulina* against ccl 4 induced liver damage in rats. Int. J. Pharm. Sci. Rev. Res..

[143] Salem S.I., Ibrahim A.K., El-Olemy K.A.a.M. (2014). Clinicopathological studies on the use of *Spirulina platensis* as a modern food supplement in alloxan-induced diabetic rats. Egyptain J. Comp. Pathol. Clin. Pathol..

[144] Abbas A.S., Shazly M.o., Ahmed K., Abdel-Mawla E., Ibrahi A.K. (2015). Therapeutic effects of *Spirulina platensis* on streptozotocin-induced diabetic rats. Egypt. J. Comp. Path Clinic Path.

[145] Brito V., da Rocha J., Puntel G., da Luz S., Barbosa N., de Carvalho N., Folmer V. (2011). Inhibition of δ-aminolevulinate dehydratase is not closely related to the development of hyperglycemia in alloxan-induced diabetic mice. Exp. Toxicol. Pathol. Off. J. Ges. Fur Toxikol. Pathol..

[146] Khan M., Shobha J., Mohan I., Rao Naidu M., Prayag A., Kutala V. (2006). *Spirulina* attenuates cyclosporine-induced nephrotoxicity in rats. J. Appl. Toxicol. JAT.

[147] Avdagić N., Cosović E., Nakas-Ićindić E., Mornjaković Z., Zaciragić A., Hadzović-Dzuvo A. (2008). *Spirulina platensis* protects against renal injury in rats with gentamicin-induced acute tubular necrosis. Bosn. J. Basic Med. Sci..

[148] Doumas B., Bayse D., Carter R., Peters T., Schaffer R. (1981). A candidate reference method for determination of total protein in serum. I. Development and validation. Clin. Chem..

[149] Arise R.O., Ganiyu A.I., Oguntibeju O., Oguntibeju O. (2014). Lipid Profile, Antidiabetic and Antioxidant Activity of *Acacia ataxacantha* Bark Extract in Streptozotocin-Induced Diabetic Rats. Antioxidant-Antidiabetic Agents and Human Health.

[150] Alderson N., Chachich M., Frizzell N., Canning P., Metz T., Januszewski A., Youssef N., Stitt A., Baynes J., Thorpe S. (2004). Effect of antioxidants and ACE inhibition on chemical modification of proteins and progression of nephropathy in the streptozotocin diabetic rat. Diabetologia.

[151] Senthilkumar R., John S.A. (2008). Hypoglycemic activity of marine cyanobacteria in alloxan-induced diabetic rats. Pharmacologyonline.

[152] Venkataraman L.V. (1998). Spirulina: Global Reach of a Health Care product.

[153] Belay A. (2002). The Potential Application of *Spirulina* (*Arthrospira*) as a Nutritional Health and Therapeutic Supplement in Health Management. J. Am. Nutraceutical Assoc..

[154] Parameshwar S., Srinivasan K.K., Rao C.M. (2002). Oral antidiabetic activities of different extracts of *Caesalpinia bonducella* Seed Kernels. Pharm. Biol..

[155] Soon Y., Tan B. (2002). Evaluation of the hypoglycemic and anti-oxidant activities of *Morinda officinalis* in streptozotocin-induced diabetic rats. Singap. Med. J..

[156] Abdel-Raouf N., Al-Enazi N.M., Ibraheem I.B.M., Al-Harbie R.M. (2015). Antibacterial and anti-hyperlipidemic activities of the brown alga *Hormophysa cuneiformis* from Ad Dammam Seashore. J. Appl. Pharm. Sci..

[157] American Diabetes Association (2010). Diagnosis and Classification of Diabetes Mellitus. Diabetes Care.

[158] Valado A., Pereira M., Amaral M., Cotas J., Pereira L. (2022). Bioactivity of carrageenans in metabolic syndrome and cardiovascular diseases. Nutraceuticals.

[159] Shirwaikar A., Rajendran K., Dinesh Kumar C., Bodla R. (2004). Antidiabetic activity of aqueous leaf extract of *Annona squamosa* in streptozotocin-nicotinamide type 2 diabetic rats. J. Ethnopharmacol..

[160] Mir S.H., Baqui A., Baghat R.C., Darzi M.M., Shah A.W. (2008). Biochemical and histomorphological study of streptozotocin-induced diabetes mellitus in rabbits. Pak. J. Nutr..

[161] Ebuehi O.A.T., Ajuluchukwu A.E., Afolabi O.T., Akinwande A.I. (2010). Oxidative stress in Alloxan--induced diabetes in female and male rats. Adv. Med. Dent. Sci..

[162] Cardoso S., Pereira O., Seca A., Pinto D., Silva A. (2015). Seaweeds as preventive agents for cardiovascular diseases: From nutrients to functional foods. Mar. Drugs.

[163] Zhou J., Zhou S., Zhang K., Tang J., Guang L., Ying Y., Xu Y., Zhang L., Li D. (2008). Chronic effects of berberine on blood, liver glucolipid metabolism and liver PPARs expression in diabetic hyperlipidemic rats. Biol. Pharm. Bull..

[164] Aboonabi A., Rahmat A., Othman F. (2015). Effect of pomegranate on histopathology of liver and kidney on generated oxidative stress diabetic induced rats. J. Cytol. Histol..

[165] Nuhu A.A. (2013). *Spirulina* (*Arthrospira*): An Important Source of Nutritional and Medicinal Compounds. J. Mar. Biol..

[166] Abdel-Daim M., El-Bialy B., Rahman H., Radi A., Hefny H., Hassan A. (2016). Antagonistic effects of *Spirulina platensis* against sub-acute deltamethrin toxicity in mice: Biochemical and histopathological studies. Biomed. Pharmacother. Biomed. Pharmacother..

[167] El-Baky H.H.A., El-Baz F.K., El-Baroty G.S. (2009). Production of phenolic compounds from *Spirulina maxima* microalgae and its protective effects in vitro toward hepatotoxicity model. Afr. J. Pharm. Pharmacol..

[168] Abdel-Daim M.M. (2014). Pharmacodynamic interaction of *Spirulina platensis* with erythromycin in Egyptian Baladi bucks (Capra hircus). Small Rumin. Res..

[169] Nugent D.A., Smith D.M., Jones H.B. (2008). A Review of Islet of Langerhans Degeneration in Rodent Models of Type 2 Diabetes. Toxicol. Pathol..

[170] Abdel-Daim M., Farouk S., Madkour F., Azab S. (2015). Anti-inflammatory and immunomodulatory effects of *Spirulina platensis* in comparison to *Dunaliella salina* in acetic acid-induced rat experimental colitis. Immunopharmacol. Immunotoxicol..

